# Microglial MS4A4A Protects against Epileptic Seizures in Alzheimer's Disease

**DOI:** 10.1002/advs.202417733

**Published:** 2025-05-11

**Authors:** Meng Jiang, Qingqing Li, Jianhui Chen, Ruochong Li, Jun Yao, Yong Hu, Haizheng Zhang, Lixin Cai, Maoguo Luo, Yu Sun, Wenwen Zeng

**Affiliations:** ^1^ Institute for Immunology and School of Basic Medical Sciences and Beijing Key Laboratory of Immunological Research of Allergy (LIRA) Tsinghua University Beijing 100084 China; ^2^ School of Life Sciences Tsinghua University Beijing 100084 China; ^3^ ENO Bio mRNA Innovation Institute Shenzhen Rhegen Biotechnology Co. Ltd Shenzhen 518000 China; ^4^ Pediatric Epilepsy Center Peking University First Hospital Beijing 100034 China; ^5^ SXMU‐Tsinghua Collaborative Innovation Center for Frontier Medicine Taiyuan 030001 China; ^6^ Tsinghua‐Peking Center for Life Sciences Beijing 100084 China

**Keywords:** Alzheimer's disease, epilepsy, microglia, MS4A4A, phagocytosis

## Abstract

Alzheimer's disease (AD) is a predominant neurodegenerative disorder worldwide, with epileptic seizures being a common comorbidity that can exacerbate cognitive deterioration in affected individuals, thus highlighting the importance of early therapeutic intervention. It is determined that deletion of *Ms4a4a*, an AD‐associated gene, exacerbates seizures in amyloid β (Aβ)‐driven AD mouse model. *MS4A4A* is significantly upregulated in brain lesions in patients with epilepsy. Single‐cell sequencing reveals that *MS4A4A* is highly expressed in microglia within these lesions, linked to enhanced phagocytic activity. Mechanistic investigation delineates that deletion of *Ms4a4a* impairs microglial phagocytosis, accompanied by diminished calcium influx and disruptions in mitochondrial metabolic fitness. The cytosolic fragment of *Ms4a4a* is anchored to the cytoskeletal components, supporting its critical role in mediating phagocytosis. Induction of *Ms4a4a* through central delivery of LNP‐*Il4* alleviates seizure conditions. Collectively, these findings identify *Ms4a4a* as a potential therapeutic target for managing seizures in AD treatment.

## Graphical abstract

This study has unveiled significant new insights into the role of MS4A4A in Alzheimer's disease‐related epilepsy, highlighting its impact on microglial phagocytosis, mitochondrial metabolism, and cytoskeleton, and demonstrating its therapeutic potential in epilepsy management.

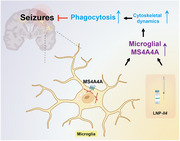

## 1 Introduction

Alzheimer's disease (AD) is a prevalent neurodegenerative disorder globally,^[^ ^1^ ^]^ characterized by progressive memory deterioration and the formation of amyloid β (Aβ) plaques and tau tangles.^[^ ^2^ ^]^ Notably, epilepsy stands out as a significant comorbidity in AD, marked by recurrent, spontaneous seizures, affecting over 20% of patients.^[^ ^3^, ^4^, ^5^, ^6^, ^7^ ^]^ Mirroring the clinical observations in patients with AD, the mouse models of AD, such as *APP/PS1* mice expressing mutant amyloid precursor protein and presenilin 1 (APPswe/PS1dE9), as well as the five times familial AD (5×FAD) mice, exhibit a high prevalence of seizures which exacerbate cognitive deficits.^[^ ^8^, ^9^, ^10^, ^11^ ^]^ In patients with AD, the comorbidity of seizures is correlated with a more pronounced deterioration of cognitive functions, underscoring the potential for early therapeutic intervention.^[^ ^8^, ^12^, ^13^ ^]^ However, the underlying mechanisms regulating the development of seizures in AD remain inadequately understood, which impedes advancements in translational research.

Previous investigation into the genetic foundations of Early Onset AD (EOAD) has yielded valuable insights into its pathophysiology, particularly regarding the roles of APP and presenilins in amyloid deposition.^[^ ^14^, ^15^, ^16^ ^]^ Over the past few decades, Genome‐Wide Association Studies (GWAS) have significantly enriched and reshaped our understanding of the genetic landscape of AD.^[^ ^17^ ^]^ Notably, various susceptibility loci have been identified, including members of the MS4A gene family,^[^ ^18^, ^19^, ^20^ ^]^ in late‐onset AD (LOAD), which accounts for over 95% of AD cases. These studies suggest that a complex intercellular mechanism underlies AD pathology.^[^ ^17^ ^]^ In particular, the discovery and characterization of variants associated with the triggering receptor expressed on myeloid cells 2 (TREM2)^[^ ^21^, ^22^, ^23^, ^24^, ^25^ ^]^ have emphasized the critical role of microglia^[^ ^26^, ^27^, ^28^, ^29^ ^]^ in AD pathogenesis. The activation state of microglia is emerging as a key factor in the initiation and progression of AD.

*MS4A4A* belongs to the *MS4A* gene family, which features four potential membrane‐spanning domains along with N‐terminal and C‐terminal cytoplasmic domains.^[^ ^30^, ^31^, ^32^ ^]^ Some family members, such as MS4A2 (FcεRIβ) and MS4A1 (CD20), have been characterized for their roles in mast cell and B cell signaling, respectively.^[^ ^32^ ^]^ Studies on MS4A4A have unveiled its function in enhancing PLCγ1 signaling in FcεRI receptor pathways in mast cells.^[^ ^33^ ^]^ In addition, *Ms4a4a* gene is expressed in macrophages during their differentiation and polarization, playing a crucial role in dectin‐1‐dependent activation of natural killer (NK) cells, which is vital for resisting tumor metastasis.^[^ ^34^ ^]^ Moreover, MS4A4A promotes alternatively activated macrophage polarization and contributes to CD8^+^ T cell dysfunction.^[^ ^35^ ^]^ Interestingly, more recent study has shown that its expression is correlated with lipid metabolism in AD.^[^ ^36^ ^]^ Single‐nucleus transcriptomic profiling of brains from patients with AD has found that protective variants increase *MS4A4A* expression, while risk variants lead to its suppression.^[^ ^36^ ^]^ The findings together establish the correlative relationship of MS4A4A with cellular energetic feature and AD pathology. More importantly, they have called for the elucidation of the precise role of MS4A4A in AD, particularly regarding its potential as a therapeutic target.

Here, we initiated our research by exploring whether MS4A4A might contribute to AD pathology. We uncovered that genetic ablation of *Ms4a4a* significantly increased the lethality of *APP/PS1* mice attributed to worsened epileptic seizure. An upregulation of *MS4A4A* was found in both epileptic patients and mouse models of seizures. We, therefore, generated the mice with conditional deletion of *Ms4a4a* in microglia which displayed exacerbated seizure conditions. Single‐cell analysis of brains from epileptic patients indicated elevated *MS4A4A* expression in regions of brain lesions was associated with heightened phagocytic activity. Mechanistic studies demonstrated that MS4A4A facilitates phagocytosis through its interaction with the cytoskeletal machinery. Overall, our findings have revealed MS4A4A as a critical regulator of microglial phagocytic capacity, which functions to ameliorate seizure conditions associated with AD.

## 2 Results

### 2.1 Loss of *Ms4a4a* Exacerbates Epileptic Death in Aβ‐Driven AD Mouse Model

**Figure 1:**
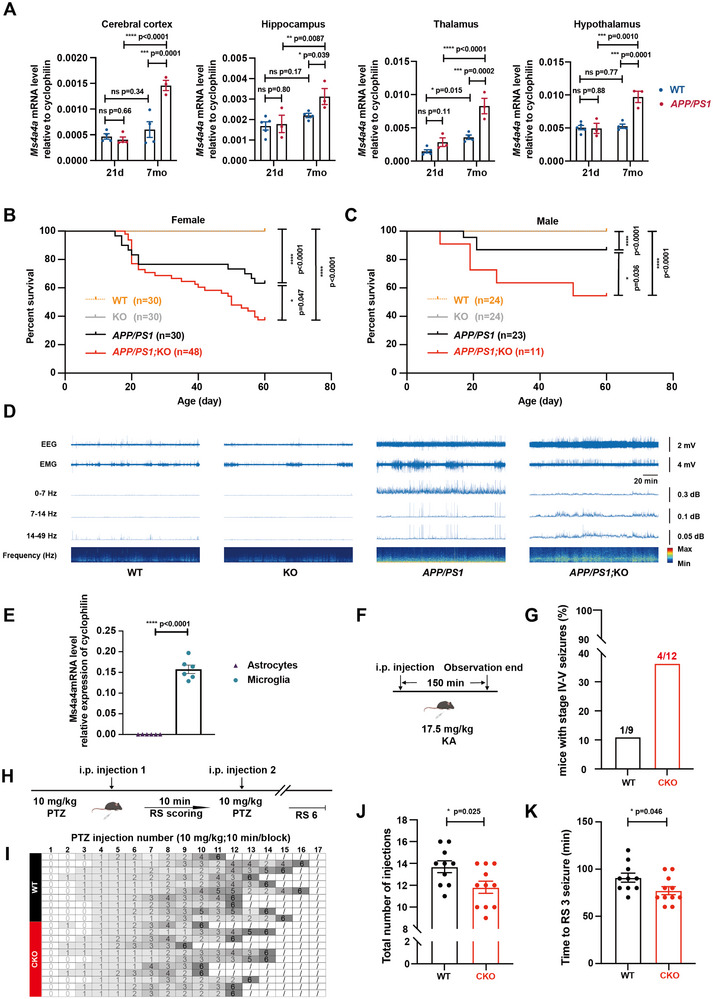
Loss of *Ms4a4a* exacerbates epileptic death in Aβ‐driven AD mouse model. A) Relative transcript levels of *Ms4a4a* in cerebral cortex, hippocampus, thalamus, and hypothalamus of 21 days and 7 months in WT and *APP/PS1* mice determined by RT‐qPCR. *n* ≥ 3. B,C) Percent survival of mouse populations of the indicated genotypes as a function of age (postnatal day) for females and males. *n* = 11–48. D) WT and KO mice showing normal EEG activity (left), *APP/PS1* and *APP/PS1;*KO mice exhibiting generalized epileptiform (interictal) spike discharges in the frequency ranges of 0–7, 7–14, and 14–49 Hz (right). *n* = 2. E) Expression levels of *Ms4a4a* mRNA in cultured primary microglia and astrocytes from WT mice determined by RT‐qPCR. *n* ≥ 4. F) The timeline showing the experimental design of the KA injection. G) The proportion of stage IV–V seizures of WT and CKO mice injected by KA within 150 min. *n* ≥ 9. H) The timeline showing the experimental design of the PTZ injection. I) The grayscale table showing behavioral seizures score of WT and CKO group. J) Quantitative analysis showing reduced total injection numbers to death in CKO. K) Quantitative analysis showing reduced time to the onset of RS 3 seizure in CKO. *n* ≥ 10. Data are presented as mean ± SEM. *p*‐values were calculated by two‐way ANOVA (A), log‐rank test (B and C) and two‐tailed, unpaired Student's *t*‐test (E, J, and K), ns *p* > 0.05, *p ≤ 0.05, **p ≤ 0.01, ***p ≤ 0.001, ****p ≤ 0.0001.

To unravel the potential function of *Ms4a4a* in AD, we utilized the *APP/PS1* transgenic mouse, an Aβ‐driven AD mouse model.^[^ ^37^, ^38^, ^39^ ^]^ We initially evaluated the expression levels of *Ms4a4a* in WT and *APP/PS1* mice at 21 days and 7 months of age. At 7 months of age, a significant upregulation of *Ms4a4a* was observed in the cerebral cortex, hippocampus, thalamus, and hypothalamus of *APP/PS1* mice compared to WT mice, during the progressive phase of AD pathology (**Figure** **1**A). This observation aligns with clinical findings of heightened *MS4A4A* expression across multiple brain regions in human AD patients.^[^ ^40^ ^]^ We then profiled its presence across various brain regions in WT mice from postnatal day 1 (P1) to postnatal day 84 (P84). Interestingly, *Ms4a4a* exhibited a negligible presence in the cerebral cortex of neonatal pups, only to surge significantly, culminating in a 17‐fold increase by P70 relative to P1 (Figure S1A, Supporting Information). In the hippocampus, *Ms4a4a* expression was notably elevated by P28, achieving a fourfold increase by P70 (Figure S1B, Supporting Information).

Subsequently, we generated a mouse line with the targeted deletion of *Ms4a4a* (Figure S1C, Supporting Information), and knockout efficiency was confirmed via RT‐qPCR analysis (Figure S1D, Supporting Information). By crossing these *Ms4a4a* knockout mice with *APP/PS1* transgenic mice, we successfully established a cohort with four distinct genotypes: *Ms4a4a^+/+^* (WT), *Ms4a4a^−/−^* (KO), *APP/PS1;Ms4a4a* ^+/+^ (*APP/PS1*), and *APP/PS1;Ms4a4a* ^−/−^ (*APP/PS1;*KO).

Intriguingly, during the breeding phase we observed that both *APP/PS1* and *APP/PS1;*KO mice displayed an increased incidence of premature mortality.^[^ ^41^ ^]^ Survival analysis from P0 to P60 revealed an early mortality peak between 2.5 and 3.5 weeks in *APP/PS1* mice, a phenomenon exacerbated by knocking out *Ms4a4a* (Figure 1B,C). The survival curves of *APP/PS1* and *APP/PS1;*KO mice diverged significantly during this period, without discernible gender bias. Knockout of *Ms4a4a* alone did not precipitate early mortality. It is noteworthy that the mortality peak coincided with the phase of increasing expression of *Ms4a4a*. Video monitoring, corroborated by electroencephalography (EEG) and electromyography (EMG) recordings, pointed to fatal epilepsy as the cause of the unexpected deaths in these mice (Video S1, Supporting Information, and Figure 1D). In contrast, mice with only the deletion of *Ms4a4a* did not exhibit epileptic activity, which prompts further investigation into the precise role of *Ms4a4a* in epilepsy initiation and progression.

Moreover, we validated the significantly high expression of *Ms4a4a* in microglia from WT mouse brains (Figure 1E), aligning with the high expression of *MS4A4A* in microglia observed in AD patients.^[^ ^40^ ^]^ In addition to microglia, we also compared the expression of *Ms4a4a* in peritoneal macrophages, alveolar macrophages and bone marrow‐derived macrophages (BMDMs) from WT mice. We found that *Ms4a4a* expression was notably high in both microglia and BMDMs, but rather lower in alveolar or peritoneal macrophages (Figure S1E, Supporting Information), suggesting a cell‐type specific regulatory role. We, therefore, developed a mouse line, *P2ry12^CreERT2^*, with Cre recombinase specifically expressed in microglia (Figure S1F, Supporting Information), as verified by immunofluorescence (Figure S1G, Supporting Information). In addition, we generated a verified mouse line with conditional *Ms4a4a* alleles (*Ms4a4a^fl/fl^*) (Figure S1H,I, Supporting Information) and crossed it with *P2ry12^CreERT2^* to selectively delete *Ms4a4a* in microglia (*P2ry12^CreERT2^;Ms4a4a^fl/fl^*, CKO), with deletion efficiency confirmed by RT‐qPCR (Figure S1J, Supporting Information). The residual amount of mRNA was possibly derived from other cell types, as shown by single‐cell sequencing analysis (Figure S3A,D, Supporting Information)

To validate our findings, we used kainic acid (KA) and pentylenetetrazole (PTZ) to induce seizures in mice. KA, an excitatory neurotransmitter receptor agonist, and PTZ, an inhibitory neurotransmitter receptor antagonist, were administered. Our results showed that, within 2 h post‐intra‐peritoneal KA injection, a higher proportion of CKO mice (4 out of 12) exhibited severe seizure behavior (score of 4–5) compared to the control group (1 out of 9) (Figure 1F,G). Moreover, CKO mice required fewer injections and less time to reach Racine Scale 3 (RS 3), a severe grade of seizure, during the PTZ injection protocol (Figure 1H–K). Similar results were observed in the global KO mice (Figure S1K,L, Supporting Information).

These findings are in concordance with our observations in *APP/PS1* and *APP/PS1*;KO mice, where the absence of *Ms4a4a* exacerbated the premature mortality of AD mice. In conclusion, our study has uncovered a potential link between AD and MS4A4A likely mediated through its association with epilepsy, suggesting that MS4A4A may play a protective role in the onset of epilepsy. We reasoned that the absence of MS4A4A alone does not manifest overt physiological defects, possibly because these defects become more pronounced when superimposed upon the AD pathological context. Those results implicate the intricate interplay between MS4A4A, epilepsy, and AD.^[^ ^42^, ^43^ ^]^

### 2.2 Expression of *MS4A4A* Is Upregulated in Lesions of Patients with Epilepsy

**Figure 2:**
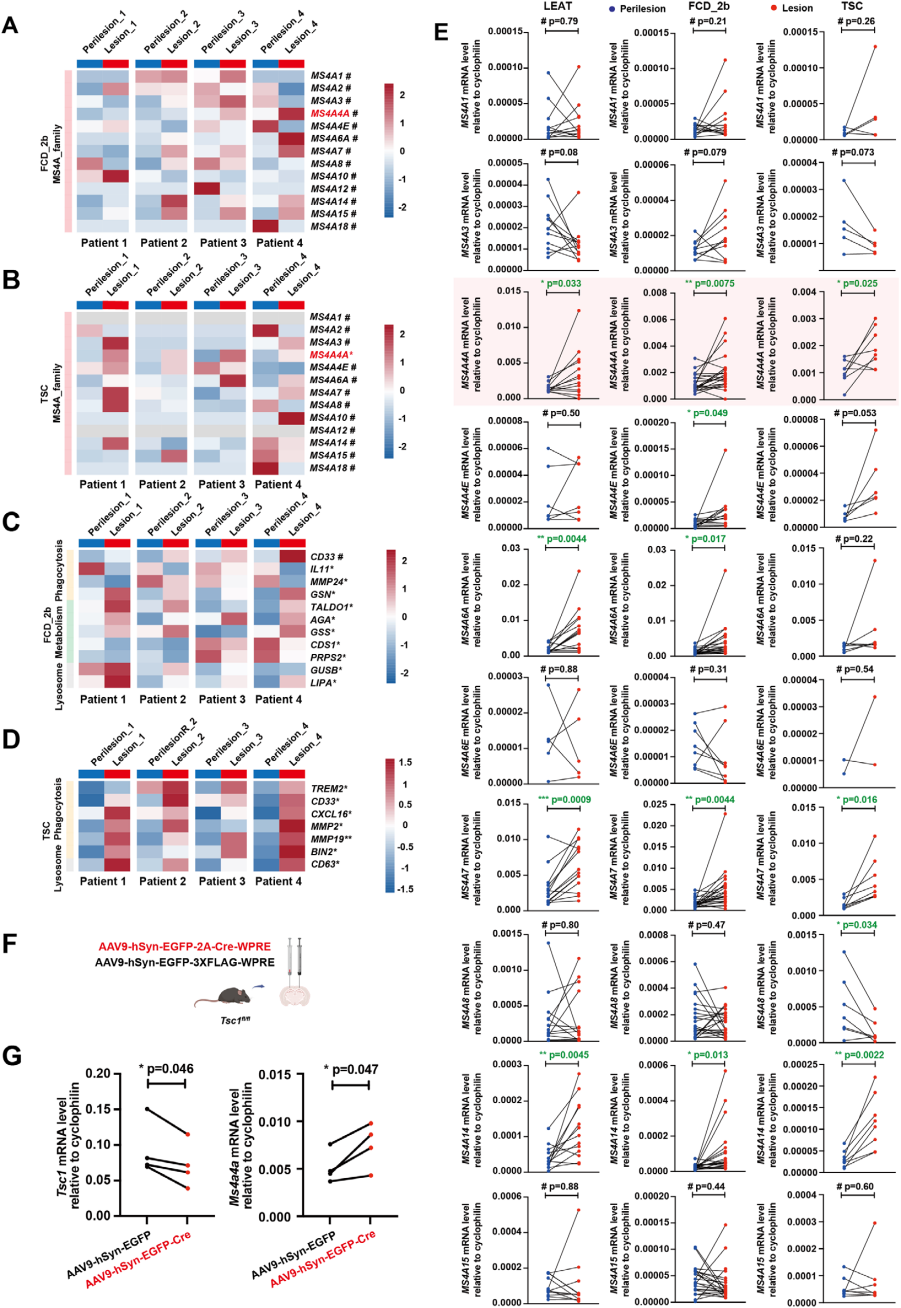
Expression of *MS4A4A* is upregulated in lesions of epilepsy patients. The heatmaps displaying the expression levels of *MS4A* family in the bulk RNA‐seq of A) FCD_2b seizure patients and B) TSC seizure patients. *n* = 4. The heatmaps displaying the expression levels of the genes related to phagocytosis and other processes in the bulk RNA‐seq of C) FCD_2b seizure patients and D) TSC seizure patients. *n* = 4. E) Relative transcript levels of *M*S*4A* family genes in lesions and perilesional tissues of LEAT, FCD_2b, and TSC patients verified by RT‐qPCR. *n* ≥ 8. F) Scheme for injecting AAV9‐hSyn‐EGFP‐2A‐Cre‐WPRE (AAV9‐hSyn‐EGFP‐3×FLAG‐WPRE as control) into *Tsc1^fl/fl^* mice. G) Relative transcript levels of *Tsc1* and *Ms4a4a* around injection site determined by RT‐qPCR. Parenchymal stereotaxic injection was conducted two weeks before RT‐qPCR. *n* = 4. Data are presented as mean ± SEM. *p*‐values were calculated by two‐tailed, paired Student's *t*‐test (A–E and G), #*p* > 0.05, *p ≤ 0.05, **p ≤ 0.01, ***p ≤ 0.001.

To substantiate our hypothesis regarding the interplay between *MS4A4A* and epilepsy, we undertook transcriptome sequencing on lesional and perilesional tissues from patients afflicted with focal cortical dysplasia type IIb (FCD_2b) and tuberous sclerosis complex (TSC) epilepsy.^[^ ^44^, ^45^, ^46^, ^47^ ^]^ The heatmaps delineate the expression profile of the *MS4A* family within microglia (**Figure** **2**A,B). In lesional tissues, *MS4A4A* was significantly upregulated in TSC patients and showed an upregulated trend in FCD_2b patients compared to controls. This pattern was accompanied by significantly differential expression across numerous genes associated with microglial phagocytosis and metabolic processes (Figure 2C,D).

Subsequently, we used RT‐qPCR to ascertain the expression levels of the *MS4A* family in clinical samples from three distinct epilepsy patient groups: long‐term epilepsy associated tumor (LEAT), FCD_2b, and TSC (Figure 2E). Our results revealed a marked upregulation of *MS4A4A, MS4A6A, MS4A7*, and *MS4A14* in lesional tissues of FEAT epilepsy patients relative to perilesional tissues. Comparable increases were noted for *MS4A4A, MS4A4E, MS4A6A, MS4A7*, and *MS4A14* in FCD_2b patients, and for *MS4A4A, MS4A7*, and *MS4A14* in TSC patients. Notably, *MS4A4A* was significantly upregulated across all three epilepsy types.

To further validate this observation, we selected TSC epilepsy, characterized by a clear genetic etiology, for validation in murine models. We administered an AAV virus carrying a Cre recombinase with neuron‐specific expression (AAV9‐hSyn‐EGFP‐2A‐Cre‐WPRE) into the cerebral cortex of *Tsc1^fl/fl^* mice (Figure 2F). This approach successfully induced deletion of *Tsc1*, leading to a pronounced increase in *Ms4a4a* expression (Figure 2G).

In summary, a significant elevation of *MS4A4A* expression has been observed in brain lesions of epilepsy patients with diverse etiologies, including FEAT, FCD_2b, and TSC mutations. Similarly, TSC mutations‐induced upregulation of *Ms4a4a* have been corroborated in mice. In addition, the absence of MS4A4A has been shown to intensify fatal epilepsy in AD transgenic mice. Collectively, the results support that the correlation between MS4A4A and AD was due to the contribution of MS4A4A to epilepsy.

### 2.3 High Expression of the *MS4A* Family Is Associated with Enhanced Phagocytic Capacity

**Figure 3:**
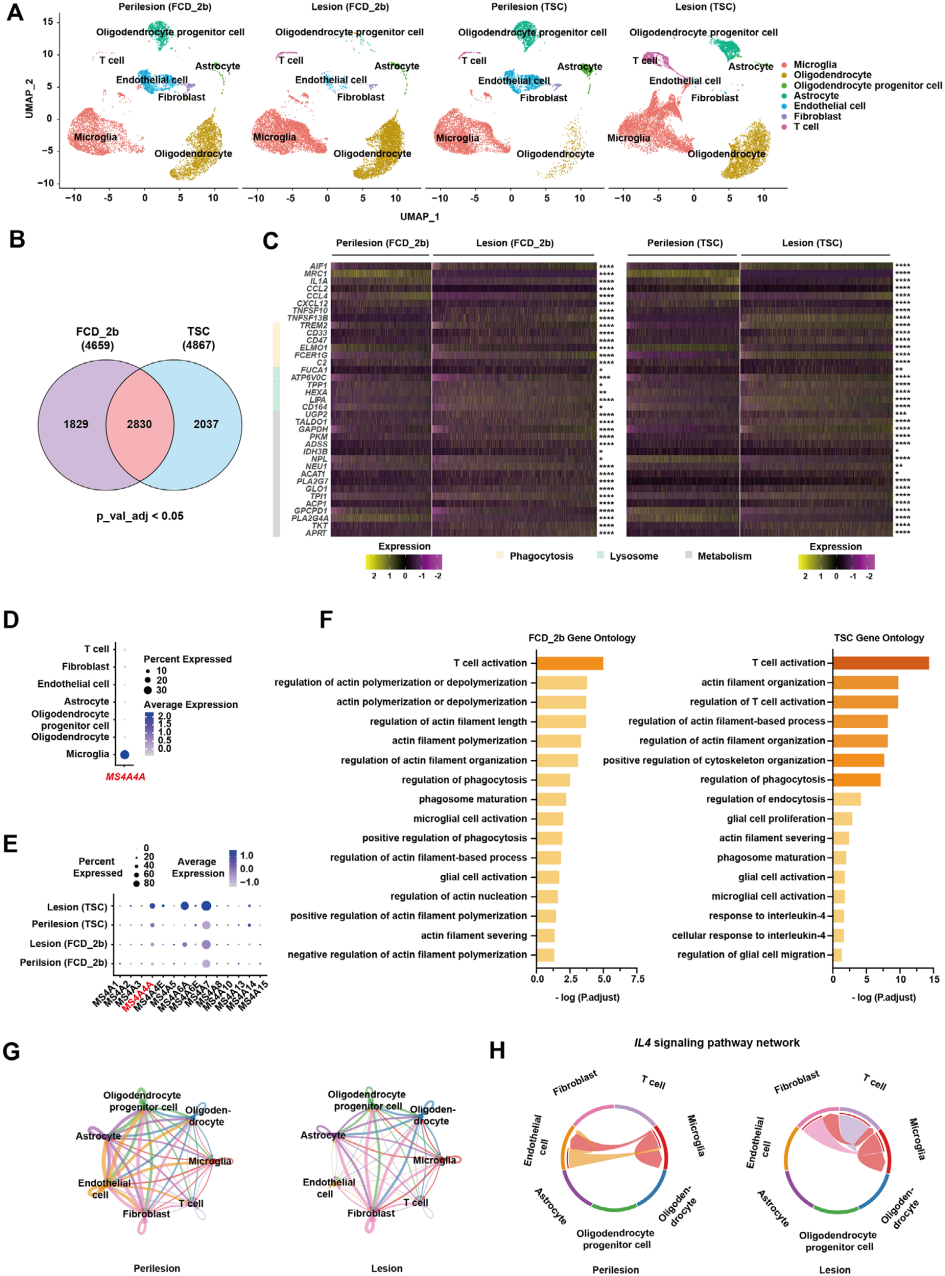
High expression of the *MS4A* family is associated with enhanced phagocytic capacity. A) t‐distributed stochastic neighbor embedding (t‐SNE) plot of seven clusters. B) Venn diagram showing the number of significantly differentially expressed genes between lesional and perilesional tissues in FCD_2b and TSC patients. p_val_adj < 0.05. C) Heatmap depicting the relative expression of selective inflammatory genes, phagocytosis genes, and metabolic genes in microglia of FCD_2b and TSC patients. The star on the right indicates the *p*‐values between lesional and perilesional microglia in FCD_2b and TSC patients. D) Dotplot showing the expression of *MS4A4A* across seven clusters and E) the expression profile of the *MS4A* family in microglia. F) Bar chart showing selectively enriched GO terms of lesional and perilesional tissues in FCD_2b and TSC. G) Network diagram displaying the intercellular communication between cell types in TSC. Each line representing the ligands expressed by the cell population, then the line connected to the cell types expressing the cognate receptors. The loops indicating autocrine circuits. The line thickness representing the number of ligand–receptor pairs. H) Circos plot showing the *IL4* signaling pathway network of lesional and perilesional tissues in TSC. *p*‐values were calculated by Wilcoxon rank sum test (B, C), *p ≤ 0.05, **p ≤ 0.01, ***p ≤ 0.001, ****p ≤ 0.0001.

To gain insights into the molecular signatures of microglia in pathological contexts, we conducted single‐cell RNA sequencing (scRNA‐seq) on lesional and perilesional samples from patients with FCD_2b and TSC. Our analysis identified seven distinct cell clusters, characterized by their expression of known marker genes (Figure S2A, Supporting Information, and **Figure** **3**A). A comparative assessment of these clusters across lesional and perilesional regions revealed notable variations in their distribution (Figure S2B, Supporting Information). Through the interaction strength of signal incoming and outgoing among cells (Figure S2C, Supporting Information), microglia showed high participation in both FCD_2b and TSC, which further emphasizes the crucial role of microglia in pathological conditions.^[^ ^48^ ^]^

In a comparative analysis of lesional and perilesional microglia in FCD_2b and TSC, 4659 and 4867 significantly differentially expressed genes were identified, respectively. Among these, 2830 genes showed significant differences in both diseases (Figure 3B). Within these 2830 genes, we pinpointed a subset that showed consistent expression changes, which were associated with inflammation, phagocytosis, lysosome, and metabolic processes in both FCD_2b and TSC (Figure 3C). *MS4A4A* was predominantly expressed in microglia and exhibited increased levels among *MS4A* family within lesional tissues (Figure 3D,E).^[^ ^32^ ^]^ Gene Ontology (GO) analysis further revealed significant enrichment of pathways associated with microglial activation, T cell activation, phagocytosis, and cytoskeletal dynamics in both FCD_2b and TSC (Figure 3F).

Focusing on TSC, we observed a notable upregulation of *IL4*, typically a T cell‐associated cytokine, within lesional tissues (Figure S2D,E, Supporting Information). This finding suggests a potential signaling mechanism underlying the upregulation of *MS4A4A* in TSC lesions.^[^ ^34^, ^49^ ^]^ A detailed analysis of signal transduction within microglia indicated a highly active state, emphasizing their critical role in crosstalk with various cell types within the brain microenvironment. Intriguingly, our analysis of cellular interactions highlighted enhanced communication among microglia and between microglia and T cells specifically within lesional areas, as well as within the *IL4* signaling network (Figure 3G,H). Besides, from microglia, fibroblasts or T cells, back to microglia, the signaling between ligand *IL4* and its receptors significantly appears in the lesional area rather than the perilesional area (Figure S2F, Supporting Information). These results hint a possible mechanism for the upregulation of *MS4A4A* in the lesional area.

### 2.4 The AD Mouse Model Amplifies the Distinctions in Microglial Functions due to the Loss of *Ms4a4a*, Specifically in Phagocytosis and Cytoskeletal Movement

To extend our investigation to a murine model, we performed scRNA‐seq on WT, KO, *APP/PS1*, and *APP/PS1;*KO mice. Our analysis identified 14 distinct cell clusters, characterized by their expression of known marker genes (Figure S3A, Supporting Information). Comparative analysis between WT and KO microglia revealed 608 significantly differentially expressed genes, whereas comparison between *APP/PS1* and *APP/PS1;*KO microglia uncovered 640 significantly differentially expressed genes (Figure S3B, Supporting Information). Among the 265 differentially expressed genes unique to group *APP/PS1* versus *APP/PS1;*KO, we identified a subset associated with inflammatory genes, phagocytosis genes, lysosome genes, and metabolic genes, indicating a role for *Ms4a4a* in modulating these processes in AD conditions (Figure S3C, Supporting Information). Consistent with what was observed in the human tissue, *Ms4a4a* expression was predominantly observed in microglia, with its expression being elevated in *APP/PS1* mice compared to WT mice (Figure S3D,E, Supporting Information). In addition, its expression was also observed in macrophages, although the total number of macrophages was much lower than that of microglia. GO analysis further confirmed significant enrichment of pathways related to microglial activation, T cell activation, phagocytosis, and cytoskeletal dynamics in *APP/PS1* compared to *APP/PS1;*KO mice (Figure S3F, Supporting Information). Cell interaction analysis underscored the pivotal role of microglia as key communicators within the brain, irrespective of their role as signal senders or receivers (Figure S3G, Supporting Information).

In summary, our single‐cell analysis across four distinct mouse genotypes has uncovered significant functional differences in microglia upon *Ms4a4a* deletion, particularly impacting phagocytosis, metabolism, and cytoskeletal dynamics. These differences are accentuated in AD models. Such disparities serve as crucial guiding directions for our subsequent experimental investigations into the functions of MS4A4A.

### 2.5 *Ms4a4a* Deficiency in Microglia Leads to Impaired Phagocytosis and Altered Calcium Signaling

**Figure 4:**
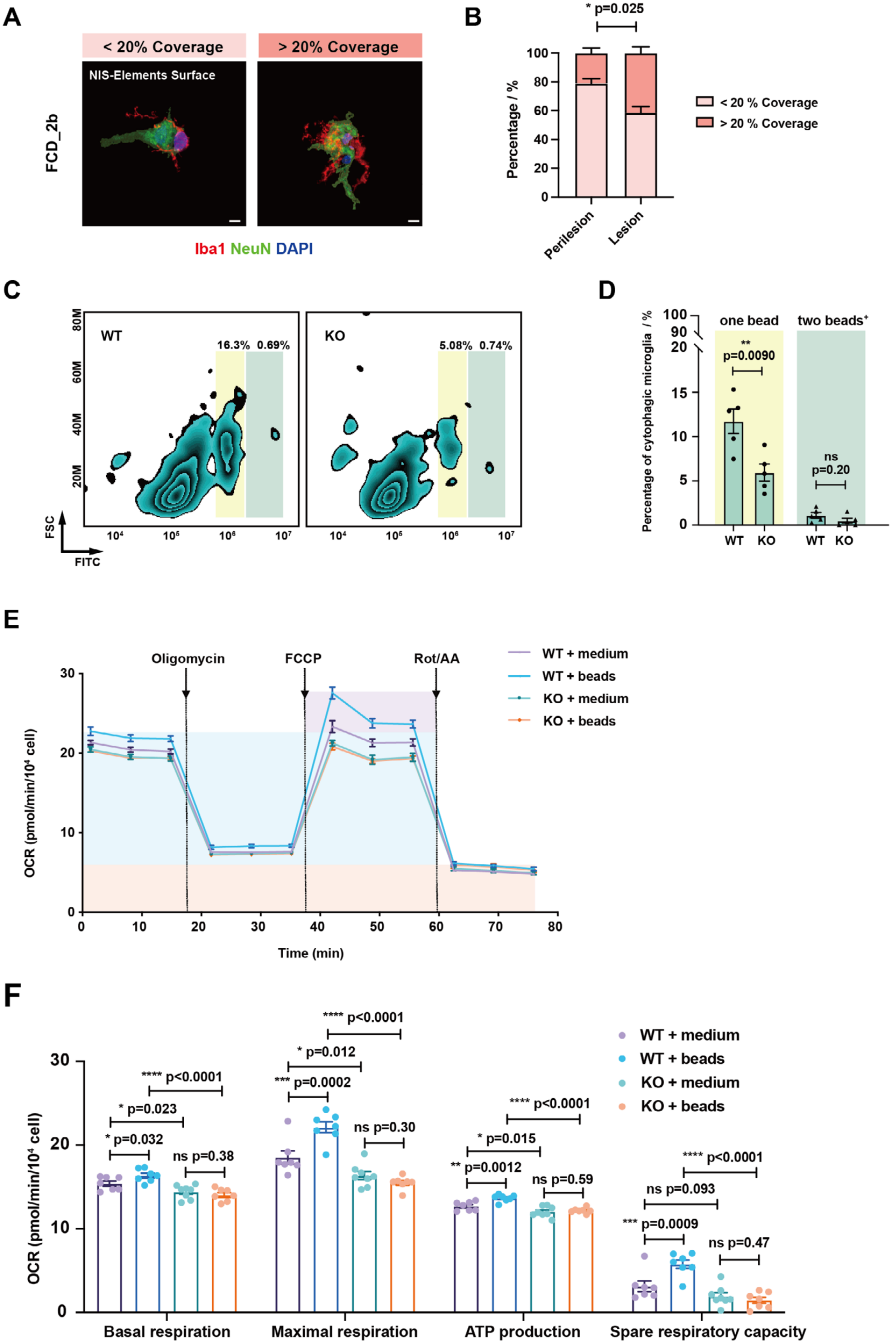
*Ms4a4a* deficiency in microglia leads to impaired phagocytosis. Representative immunofluorescence images showing Iba1 with varying coverage of NeuN in brain tissues of A) FCD_2b patients and B) the percentage of Iba1 with different NeuN coverage. Scale bars, 10 µm. Survey of over 180 microglia from 8 to 10 subfields and *n* = 4 patients/group. C) Representative flow cytometry plots of cultured primary microglia (WT and KO) after co‐culture with 6 µm fluorescent beads for 2 h. D) Phagocytic ability indicated by the proportion of 6 µm fluorescent beads inside the cultured primary microglia declined. *n* ≥ 4. E,F) Representative OCR measurements obtained from Seahorse assays, normalized to microglia intensity, for WT, WT+beads, KO, and KO+ beads groups. Arrows indicating addition of oligomycin, FCCP, and rotenone+antimycin A (Rot/AA) (E). Quantification of basal respiration, maximal respiration, ATP production, and spare respiratory capacity from Seahorse experiments depicted in (E) with four group cultured primary microglia (F). *n* ≥ 6. Data are presented as mean ± SEM. *p*‐values were calculated by two‐tailed, paired Student's *t*‐test (B), two‐tailed, unpaired Student's *t*‐test (D), and two‐way ANOVA (F), ns *p* > 0.05, *p ≤ 0.05, **p ≤ 0.01, ***p ≤ 0.001, ****p ≤ 0.0001.

Microglia are distinguished from other cell types by their robust phagocytic capabilities, a feature central to their role in immune surveillance within the central nervous system (CNS).^[^ ^50^, ^51^, ^52^ ^]^ Our single‐cell analysis consistently revealed significant differences in microglial phagocytosis in patient samples with variable *MS4A4A* expression and in mice with *Ms4a4a* knockout. This observation prompted us to closely examine the influence of MS4A4A on microglial phagocytic function. Previous research suggests that microglia may exhibit phagocytic behavior towards neurons during epilepsy onset and microglial phagocytic alterations are strongly associated with elevated susceptibility to epileptic seizures.^[^ ^53^, ^54^, ^55^, ^56^ ^]^ Using tissue immunofluorescence staining, we observed varying degrees of microglial phagocytic activity towards neurons in control and epileptic zones (**Figure** **4**A,B). This led us to investigate the impact of MS4A4A on microglial phagocytosis by culturing primary microglia from WT and KO mice and assessing their phagocytic capabilities using 6 µm beads as targets. We observed a significant decline in the phagocytic ability of *Ms4a4a*‐deficient microglia (Figure 4C,D). When other types of phagocytic substrates were added, including 1 µm beads, 2 µm beads, GFP‐fluorescent *E. coli*, and GFP‐fluorescent yeast strain H99, KO microglia consistently exhibited reduced phagocytic capacity (Figure S4A–D, Supporting Information). Therefore, we concluded that the phagocytic defect caused by *Ms4a4a* knockout is not substrate‐specific. Given that the deficiency of *Ms4a4a* led to reduced phagocytic capacity, we examined the plaque number in cerebral cortex of *APP/PS1* and *APP/PS1;*KO mice and found that knockout of *Ms4a4a* appeared to result in a higher burden of plaques in *APP/PS1;*KO mice (Figure S4E, Supporting Information).

The phagocytic process is energy demanding and closely correlated with metabolic energy. To discern the metabolic differences between WT and KO microglia during phagocytosis, we measured the oxygen consumption rate (OCR) of WT and KO microglia by using 2 µm beads (Figure 4E). We noted there was a decrease in basal, maximal respiration, and ATP production in KO groups compared with WT groups (Figure 4F). Moreover, in response to 2 µm beads, WT microglia exhibited enhanced mitochondrial capacity, while KO microglia exhibited significant deficiencies (Figure 4F), aligning with the phagocytic defects observed in KO microglia.

**Figure 5:**
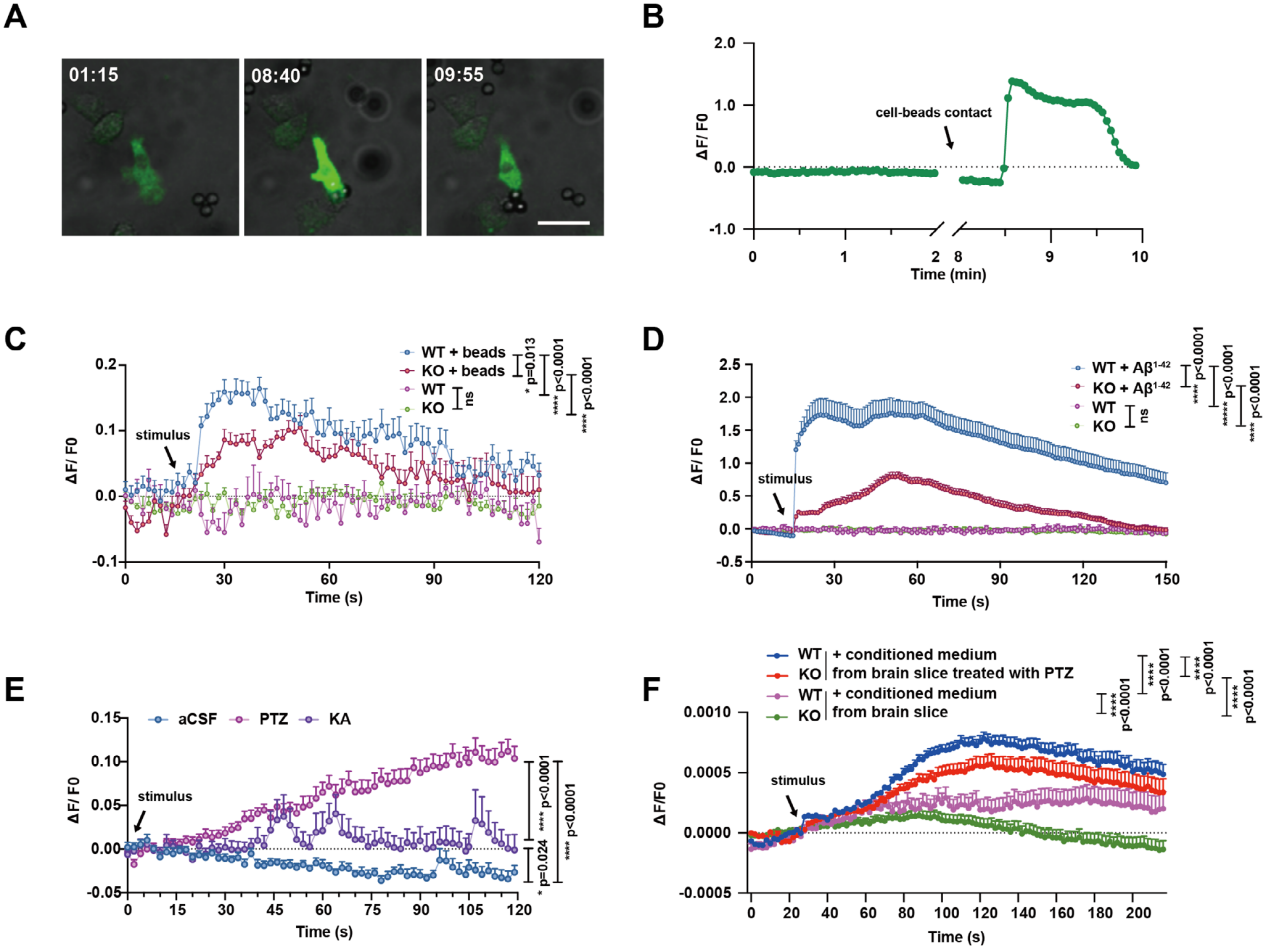
*Ms4a4a* deficiency in microglia leads to altered calcium signaling. A) Representative immunofluorescence images and B) calcium activity (Δ*F*/*F*) in the process of touching and engulfing 6 µm beads by *P2ry12^CreERT2^;Ai95* microglia in vitro. Scale bars, 25 µm. Calcium activity (Δ*F*/*F*) of WT and KO microglia indicated by calcium flux indicator Rhod2 after adding C) 6 µm beads and D) HiLyte Fluor 488 Aβ_1‐42_ separately. *n* ≥ 10. E) Calcium activity (ΔF/F) of live *P2ry12^CreERT2^;Ai95* brain slices after adding aCSF, 100 × 10^−3^ m PTZ and 100 × 10^−3^ m KA separately. *n* ≥ 10. F) Calcium activity (Δ*F*/*F*) of WT and KO microglia indicated by calcium flux indicator Rhod2 after adding supernatant collected from live brain slices stimulated with 100 × 10^−3^ m PTZ or aCSF. *n* ≥ 30. Data are presented as mean ± SEM. *p*‐values were calculated by two‐way ANOVA (C–F), ns *p* > 0.05, *p ≤ 0.05, ****p ≤ 0.0001.

While the abnormal discharge of neurons serves as the direct cause of epilepsy, the potential contributions of microglia have been recognized just recently.^[^ ^57^ ^]^ For instance, microglia are proposed as key regulators of neuronal network and related behavioral responses: they suppress neuronal activity upon activation and their ablation leads to amplified and synchronized neuronal activity, resulting in seizures.^[^ ^58^ ^]^ Our results are in align with the notion that microglia, as resident immune cells with heightened effector functions, may play a crucial role in the pathogenesis of epileptic seizures. Microglial calcium signaling, a critical surrogate indicator of phagocytic activity, is strongly engaged during epilepsy development and fluctuates with intracellular activities.^[^ ^56^, ^59^, ^60^ ^]^ To substantiate the involvement of MS4A4A in microglial phagocytosis during epilepsy pathogenesis, we generated *P2yr12;Ai95* mice to specifically track calcium flux within microglia. In the imaging experiments, we observed that microglia engaged their phagocytic function upon encountering beads as stimuli, accompanied by significant alterations in calcium signaling (**Figure** **5**A,B and Video S2, Supporting Information). Interestingly, the compromised phagocytic ability in *Ms4a4a*‐deficient microglia corresponded with reduced amplitude of calcium signaling changes, as indicated by the calcium flux indicator Rhod2, upon exposure to beads and Aβ^1‐42^ (Figure 5C,D and Video S3–S5, Supporting Information).

In parallel, ex vivo brain tissue slices from these mice were subjected to calcium imaging following stimulation with epileptic drugs. Notably, we observed significant alterations in calcium flux within microglia upon drug administration compared to artificial cerebrospinal fluid (aCSF) controls (Figure 5E and Video S6, Supporting Information). Considering the phagocytic impairments observed in KO groups, it is plausible that these defects compromise the ability to process microenvironmental changes upon abnormal neuronal activity during epileptic seizures. To investigate this, we collected the supernatant from live brain slices treated with epileptogenic drugs PTZ and applied it to the cultured primary microglia. The findings revealed that the KO group exhibited a significantly reduced amplitude of calcium flux compared to the WT group (Figure 5F). This suggests that the post‐seizure brain scavenging by microglia is dependent on MS4A4A.

We conclude that the absence of MS4A4A in microglia leads to a reduction in their phagocytic capacity, thereby impairing the engulfment function, and ultimately disrupting cerebral homeostasis. This disruption is magnified in the pathogenesis of epilepsy, as evidenced by the increased incidence of fatal seizures and reduced survival rate in *APP/PS1* mice deficient in *Ms4a4a*. The exacerbation of this imbalance during epileptic events suggests a critical role for MS4A4A in modulating the severity and potentially the prognosis of the disease.

### 2.6 MS4A4A Promotes Phagocytosis through Interacting with Cytoskeleton

**Figure 6:**
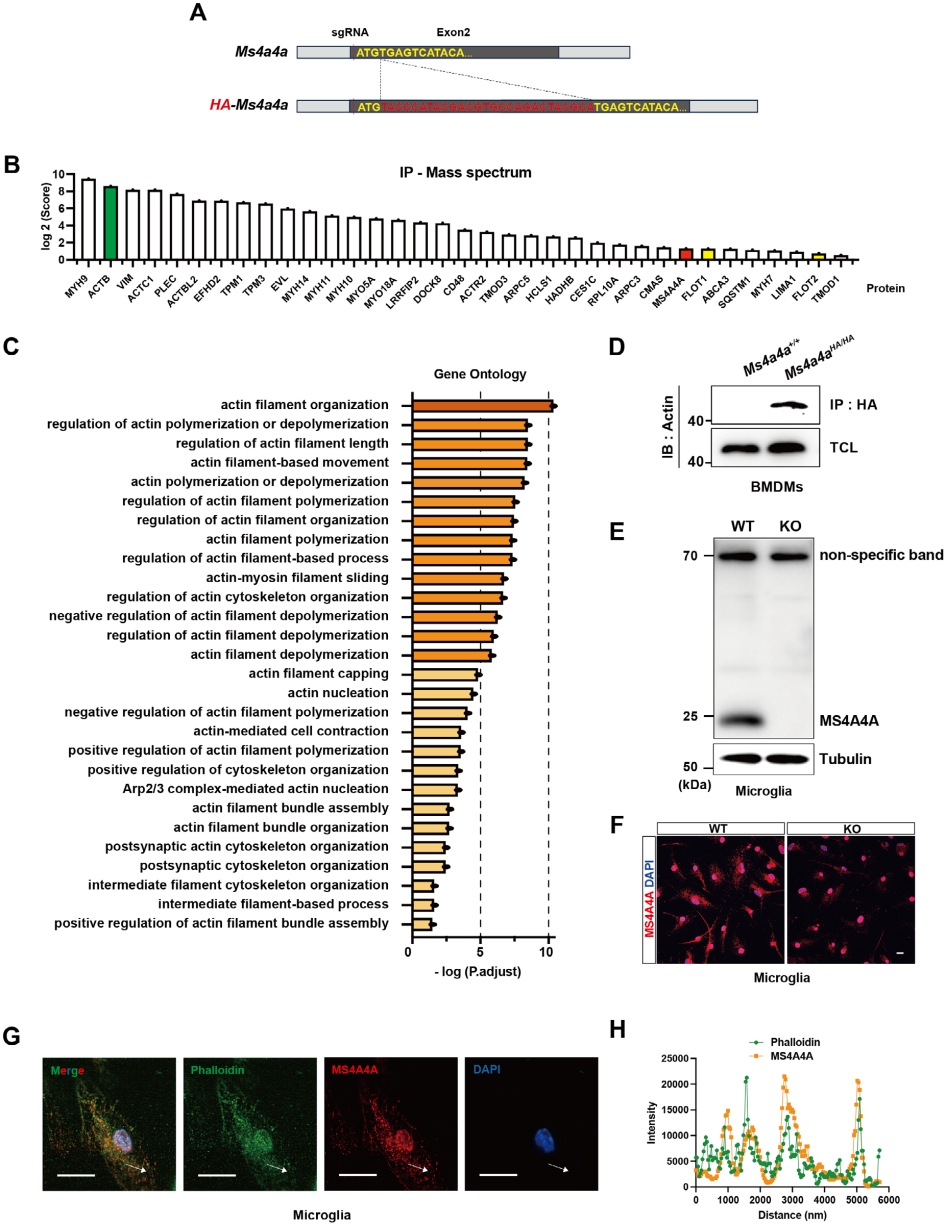
MS4A4A mediates phagocytosis and is possibly through interacting with cytoskeleton. A) Strategy of *Ms4a4a^HA/HA^* mouse. B) The ranking list of proteins interacting with MS4A4A as identified by mass spectrometry. C) GO analysis of mass spectrum results listed potential proteins interacted with MS4A4A. D) Verification of interaction between MS4A4A and Actin by co‐immunoprecipitation assay in BMDMs. E) Western blot and F) representative immunofluorescence captures showing MS4A4A expression of WT and KO microglia. Scale bar, 5 µm. G) Representative immunofluorescence captures showing the spatial position of MS4A4A and Actin stained by phalloidin in microglia. H) The histogram shows that MS4A4A colocalized with phalloidin. Scale bar, 10 µm.

To dissect the molecular underpinnings of the functional role of MS4A4A, we generated a mouse line expressing an N‐terminal HA‐tagged MS4A4A (**Figure** **6**A). We enriched HA‐MS4A4A in BMDMs through immunoprecipitation and further analyzed their interacting proteins by mass spectrometry, which shows that the proteins interacting with MS4A4A are mostly cytoskeletal proteins (Figure 6B). Further GO analysis indicates that these proteins are highly enriched in pathways such as the cytoskeleton movement (Figure 6C). These findings suggest a significant role for MS4A4A in modulating cytoskeletal architecture. Western blot (WB) analysis substantiated the interaction between MS4A4A and Actin, a key cytoskeletal component (Figure 6D). We then developed polyclonal antibodies against MS4A4A by immunizing mice with a recombinant protein containing fused fragments of N‐terminal and C‐terminal of MS4A4A. These antibodies were validated for specificity and detection efficacy using western blot and immunofluorescence (Figure 6E,F). It should be noted that, in the western blot validation of the antibody, there was a nonspecific band with a higher molecular weight, which may account for the nonspecific staining in the nucleus observed in immunofluorescence staining. Subsequent immunofluorescence staining of cultured primary microglia revealed a co‐localization of MS4A4A with Actin (Figure 6G,H). Collectively, these results support the functions of MS4A4A and Actin in the orchestration of cellular phagocytosis.

Notably, Flotillin‐1 and Flotillin‐2, two scaffold proteins known for their ability to form stable complexes,^[^ ^61^ ^]^ are significant interactors with MS4A4A according to our mass spectrometry results (Figure 6B). To validate these interactions, we employed co‐immunoprecipitation assays using MS4A4A tagged with FLAG at its C‐terminus and Flotillin‐1 tagged with Strep at its C‐terminus (Figure S5A, Supporting Information), co‐expressed in the 293F cell line. Co‐immunoprecipitation using anti‐FLAG antibodies, followed by western blot detection of both tags, confirmed the interaction between MS4A4A and Flotillin‐1 (Figure S5B, Supporting Information). Truncation assays revealed that the C‐terminal domain of MS4A4A is essential for its interaction with Flotillin‐1, while Flotillin‐1 likely interacts with MS4A4A via its N‐terminal domain (Figure S5A,B, Supporting Information).

To visualize the spatial dynamics of MS4A4A and Flotillin‐1 during phagocytosis, we performed immunofluorescence staining on cultured primary microglia challenged with 2 µm beads. Our results demonstrated a reduction in the spatial distance between MS4A4A and Flotillin‐1, along with an increased co‐localization during phagocytic engulfment, compared to non‐stimulated controls (Figure S5C,D, Supporting Information). These observations suggest physical and functional associations between MS4A4A and Flotillin‐1, mediated by their C‐terminal and N‐terminal domains, respectively, and underscore their collective involvement in microglial phagocytosis.

### 2.7 Administering *Il4* mRNA Encapsulated in Lipid Nanoparticles (LNPs) to the CNS Effectively Enhances the Tolerance to Epileptic Onset

**Figure 7:**
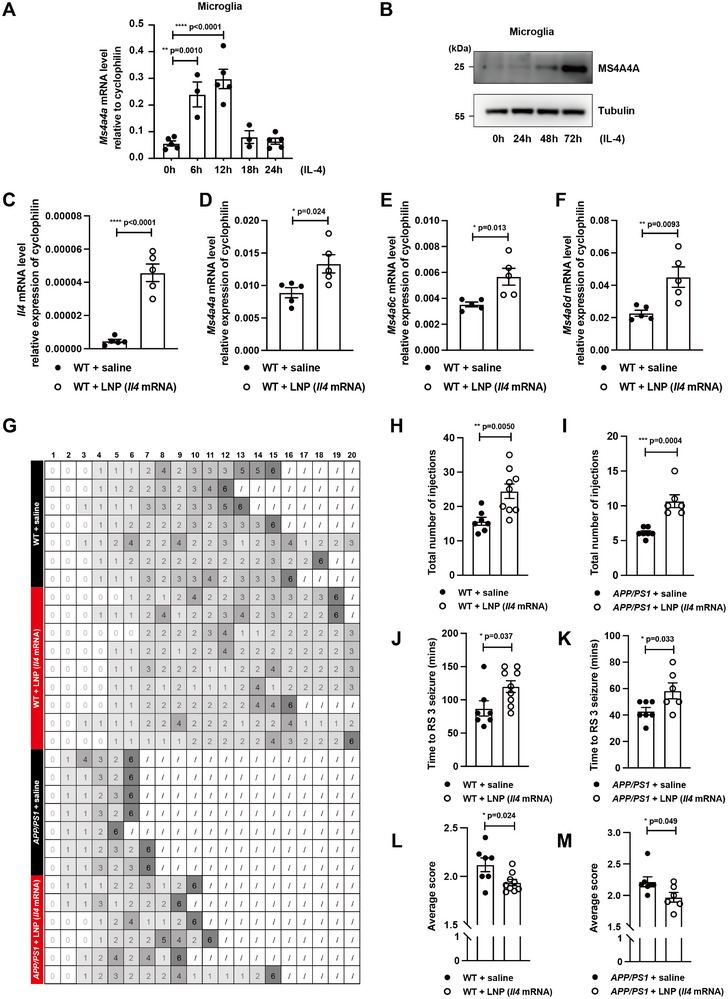
Administering *Il4* mRNA encapsulated in lipid nanoparticles (LNPs) to the CNS enhances the tolerance to epileptic onset. A) Relative transcript levels of *Ms4a4a* in microglia treated with IL‐4 for the indicated time determined by RT‐qPCR. *n* ≥ 3. B) Western blot showing MS4A4A expression in cultured primary microglia treated with IL‐4 for the indicated time. *n* = 3. C–F) Relative transcript levels of *Il4*, *Ms4a4a*, *Ms4a6b*, *Ms4a6c* in cerebral cortex of WT+saline and WT+LNP groups determined by RT‐qPCR. *n* = 5. G–M) The grayscale table showing behavioral seizure scores of the four groups (WT+saline, WT+LNP, *APP/PS1*+saline, and *APP/PS1*+LNP) after the drug‐induced seizure model was performed (G). The total injection number to death of WT groups (H) and *APP/PS1* groups (I). Time to the onset of RS 3 seizure of WT groups (J) and *APP/PS1* groups (K). Average score of WT groups (L) and *APP/PS1* groups (M). *n* ≥ 6. Data are presented as mean ± SEM. *p*‐values were calculated by one‐way ANOVA (A) and two‐tailed, unpaired Student's *t*‐test (C–F and H–M), *p ≤ 0.05, **p ≤ 0.01, ***p ≤ 0.001, ****p ≤ 0.0001.

IL‐4 has been shown to upregulate the expression of MS4A4A in macrophages,^[^ ^62^, ^63^ ^]^ and we assessed the temporal expression profiles of *Ms4a4a* in microglia across both transcriptional and translational dimensions. Notably, *Ms4a4a* mRNA exhibited a marked elevation 6 and 12 h post‐IL‐4 induction, subsequently declining by the 18‐ and 24‐h marks (**Figure** **7**A). In tandem, employing our MS4A4A antibodies we observed an ascending trend in MS4A4A protein levels at the 24, 48, and 72 h subsequent to IL‐4 induction (Figure 7B).

With the expression profile of *Ms4a4a* under IL‐4 regulation in microglia established, and backed up by our preliminary data hinting at a protective function for *Ms4a4a* in seizure resistance, we proceeded to administer *Il4* mRNA encapsulated by LNP into the murine CNS. This strategic delivery aimed to upregulate *Ms4a4a* expression and observe its therapeutic potential. RT‐qPCR analysis of the mouse cerebral cortex, conducted 6 h following LNP‐*Il4* administration, substantiated a significant upregulation in mRNA levels of *Il4*, *Ms4a4a*, and its family member *Ms4a6* (Figure 7C–F). Interestingly, the experimental group, including both WT and *APP/PS1* strains, with LNP‐*Il4* administration showed significant improvement against drug‐induced epileptic seizures compared to the control group, with both the total number of injections required for death and the time to reach RS 3 effectively prolonged (Figure 7G–K). Moreover, the group with LNP‐*Il4* injected exhibited a diminished average score in drug‐induced epileptic seizures (Figure 7L,M).

Collectively, our investigation has unveiled a viable approach to defer epileptic episodes via targeted CNS delivery, heralding promising avenues for clinical epilepsy management.

## 3 Discussion

In our research, we identified MS4A4A as a crucial regulator in the control of seizures in AD. Our findings indicate that MS4A4A is upregulated in microglia in AD mouse models, mice with epilepsy and epileptic patients, suggesting its relevance to seizure activity. Deletion of *Ms4a4a* exacerbates seizures and increases mortality in AD mouse model. In addition, *Ms4a4a* ablation leads to a more severe seizure in drug‐induced seizure models. Single‐cell sequencing analysis revealed that *MS4A4A* expression is notably high in microglia within brain lesion areas, which are associated with robust phagocytic capabilities. The deletion of *Ms4a4a* in AD mouse models led to impaired microglia phagocytosis. At the molecular level, we found that *Ms4a4a* is clustered on plasma membrane and mediates phagocytosis through anchoring to the cytoskeleton network. Interestingly, inducing MS4A4A expression by administration of IL‐4 could alleviate seizure conditions (Figure S6, Supporting Information).

AD is now recognized as a biological continuum, with early signs involving neuronal network dysfunction.^[^ ^64^ ^]^ Patients with AD with comorbid seizures had increased Aβ and tau pathology, worsened cognitive and functional scores.^[^ ^8^, ^65^, ^66^ ^]^ Past research indicates that neuronal hyperactivity contributes to plaque formation, and suppressing the hyperactivity linked to seizures ameliorates tau pathology and the associated behavioral deficits in AD mouse model.^[^ ^67^, ^68^ ^]^ Moreover, managing neuronal hyperactivity in epileptic AD mice has been shown to have a positive impact on memory deficits over time.^[^ ^8^ ^]^ The 3Tg‐AD mouse, a triple transgenic mouse model of familial AD (3×Tg‐AD mouse) that harbors mutated human APP, tau and presenilin 1 (PS1) genes shows increased incidence of audiogenic seizures compared with WT controls, and seizure susceptibility was attenuated by passive immunization with anti‐human APP/Aβ antibody (6E10).^[^ ^69^ ^]^ Furthermore, rapamycin rescues vascular, metabolic and learning deficits in apolipoprotein E4 transgenic mice with pre‐symptomatic AD.^[^ ^70^ ^]^ Chronic rapamycin restores brain vascular integrity and function through NO synthase activation and improves memory in symptomatic mice modeling AD.^[^ ^71^ ^]^ In addition, pharmacological interventions have been shown to effective to restore calcium homeostasis accompanied by normalizing brain network activity, reducing Aβ and tau pathology in AD models.^[^ ^72^ ^]^ Our findings suggest that MS4A4A act as a negative regulator of neuronal hyperactivity in the early stages of AD development. Collectively, we propose that targeting MS4A4A, either through agonistic means or by enhancing its expression in the brain, could potentially mitigate neuronal hyperactivity and thus effectively slow the progression of AD pathology.

The relationship between epilepsy and AD has garnered significant attention in recent years, with accumulating evidence pointing towards a potential bidirectional link between these two conditions. In the context of AD, it is highly possible that the chronic neuroinflammation and synaptic dysfunction associated with AD pathology could lower the seizure threshold, making individuals more susceptible to epileptic seizures. Conversely, repeated seizures in epilepsy may lead to neuronal injury and neuroinflammation, potentially accelerating the progression of AD pathology. Observational studies have suggested that epilepsy occurring before an AD diagnosis may serve as a risk factor for the development of AD.^[^ ^67^, ^73^ ^]^ This finding underscores the importance of understanding the underlying mechanisms that connect these two seemingly distinct neurological disorders.

Microglia serve as the central phagocytic cells in the brain, tasked with the critical function of waste scavenging. Recent research has underscored their indispensable role in cognitive processes, arousal, and seizure regulation. Our study indicates that impaired phagocytosis can have severe consequences on the brain neuronal network. Interestingly, deficiency in microglial TREM2 has been linked to reduced phagocytic activity and increased epilepsy,^[^ ^74^ ^]^ while increased phagocytic activity has also been associated with seizures.^[^ ^54^ ^]^ Moreover, recent studies have suggested that the *MS4A* gene family contains key modulators of soluble TREM2 (sTREM2), which has been linked to AD when present in cerebrospinal fluid (CSF).^[^ ^75^ ^]^ Structurally, TREM2 is an immunoglobulin‐like transmembrane receptor that directly regulates microglial phagocytosis and inflammation via the DAP12 signaling pathway,^[^ ^29^ ^]^ while MS4A4A belongs to the MS4A family with distinct transmembrane domains and may indirectly modulate microglial activity by influencing TREM2 stability or soluble TREM2 (sTREM2) levels.^[^ ^75^ ^]^ Functionally, *Trem2* loss‐of‐function mutations impair Aβ clearance, whereas *MS4A4A* protective variants (e.g., rs1582763) are associated with elevated sTREM2 and reduced AD risk.^[^ ^76^ ^]^ These insights collectively emphasize the necessity for a precisely regulated balance of microglial phagocytosis for maintaining brain health. However, current methods fall short of providing a noninvasive means to assess microglial states, particularly in quantifying phagocytic capacity. It appears that scavenging capacity cannot be accurately extrapolated from currently available inflammatory markers, as our study and previous research have shown.^[^ ^54^ ^]^ Developing a tool to measure microglial states, including phagocytosis, for diagnostic and therapeutic purposes would benefit precise management.

In the pathological contexts of AD or epilepsy, we have primarily concentrated on the role of microglia. However, it is crucial to acknowledge *Ms4a4a* was also expressed on the infiltrating macrophages. Previous studies, whether through sequencing or experimental approaches, have consistently demonstrated the heterogeneity in distribution and function between infiltrating macrophages and resident microglia in CNS diseases.^[^ ^77^, ^78^, ^79^, ^80^ ^]^ In addition, there is evidence suggesting that microglia, beyond maintaining CNS homeostasis and responding to injury, can also influence tissue repair and functional recovery after injury by modulating the activity of other cell types, such as macrophages.^[^ ^81^ ^]^ Therefore, the contribution of macrophages or cellular crosstalk between microglia and infiltrating warrants further exploration. Investigating the role of *Ms4a4a* in infiltrating macrophages and its potential interactions with microglia could provide valuable insights into the complex immune dynamics in the brain during chronic neurodegenerative diseases.

Our results show that MS4A4A does not show selectivity when responding to various particles in vitro. It is possible that MS4A4A adopted a shared phagocytic process to various stimuli, the underlying mechanism of which warrants further investigation. In mast cells, MS4A4A promotes the recruitment of the receptor tyrosine kinase KIT into caveolin‐1‐enriched microdomains and signaling through PLCγ1,^[^ ^82^ ^]^ and KIT‐mediated Ca^2+^ conductance promotes synergy in Ca^2+^ responses.^[^ ^33^ ^]^ In macrophages, MS4A4A and Dectin‐1 associate in lipid rafts after Zymosan engagement.^[^ ^34^ ^]^ Our data showed that MS4A4A interacts with Actin and Flotillin‐1 and potentiates calcium signaling. The functions of microglia are highly dependent on intracellular calcium signaling. More than 20 different ligands for ionotropic and metabotropic receptors have been identified to induce receptor‐mediated calcium signaling in microglia in vitro.^[^ ^83^, ^84^ ^]^ Microglia enhance their calcium signaling in various situations where phagocytosis is likely to occur, such as contact with Aβ plaques,^[^ ^85^ ^]^ or in epileptogenesis conditions.^[^ ^56^, ^86^ ^]^ Taken together, we speculate that the stacking of transmembrane domains of MS4A4A may promote the clustering effects, lipid raft molecule sorting, and co‐opt the signaling events of calcium and cytoskeletal reorganization for the formation of phagosomes and the phagocytosis process.

In addition, we observed an increased expression of *MS4A4A* in several types of patients with epilepsy. The upregulation of *MS4A4A* in lesion areas may represent a protective mechanism initiated by the body to counteract and balance the progression of epilepsy. Specifically, epileptic seizures could trigger a stress response in the nervous system, leading to increased *MS4A4A* expression in an attempt to enhance its protective functions and mitigate the damage caused by epilepsy. However, given the complexity and intractability of pathological process of epilepsy, these protective mechanisms, including the upregulation of *MS4A4A*, may only slow disease progression rather than halt it entirely. Thus, the observed increase in *MS4A4A* in lesion areas of epilepsy patients may be a result of the active defense of the body against the disease, rather than a direct cause of epilepsy onset.

Microglia could undergo significant changes in disease progression.^[^ ^36^ ^]^ On one side, microglia could be crucial in curbing epileptogenesis as direct depletion of microglia has worsen the severity of acute and chronic seizures in mice.^[^ ^87^ ^]^ On the other side, signal alteration such as missing *Ms4a4a* gene or perturbing ST2 signal axis exacerbate seizures.^[^ ^53^ ^]^ These collective findings implicate that microglial state changes might serve as a double‐edged sword for seizure conditions. For patients susceptible to seizures, the period of latency following the initial insults such as stroke or infections, with epileptogenesis persisting after the first seizure, provides a therapeutic window for modifying treatment. Altering the microglia states during this window could offer a promising approach to prevent the development of epilepsy. Looking ahead, research into key genes including *MS4A4A*, which are associated with microglial states and functions, may pave the way for the development of disease‐modifying anti‐epileptogenic medications (AEMs).^[^ ^88^ ^]^ Such advancements could extend beyond treating seizures associated with AD, potentially informing the management of epilepsy in a broader context.

Finally, *Ms4a4a* is an attractive therapeutic target given its cell‐specific expression. Its restricted presence in certain cell types, including microglia and macrophages, minimizes potential off‐target effects and enhances treatment safety and efficacy. Development of diagnostic tools, biomarkers or intervention strategy based on *Ms4a4a* is expected in AD or epilepsy pathology.

## 4 Experimental Section

### Animal Information

All the experimental procedures in mice were performed in compliance with the protocol approved by the Institutional Animal Care and Use Committee of Tsinghua University and the NIH Guide for the Care and Use of Laboratory Animals.

Animals were maintained on the 12‐h light/12‐h dark cycles, temperature (20–23 °C) and relative humidity (40%–65%) with the chow diet and water available ad libitum. All protocols used in this research were prepared before the study. All protocols were established in the lab and registered in Laboratory Animal Resources Center, Tsinghua University. Both male and female mice maintained in specific pathogen‐free conditions were utilized in the experiments. Wildtype C57BL/6 mice were purchased from Charles River International.

*APP/PS1* (JAX 004462, RRID:IMSR_JAX:004462), *Ai95* (JAX 028865, RRID:IMSR_JAX:028865), *Tsc1^fl/fl^* (JAX 038428, RRID:IMSR_JAX:038428) were purchased from the Jackson Laboratory.

KO mouse line was generated by disruption of exon 2. Cas9 mRNA and sgRNA (5′‐sgRNA: 5′‐ TATATGTGAATTCGCATTCATGG‐3′) were delivered into C57BL/6 mouse zygotes via microinjection. The resulting offspring were screened by PCR genotyping, DNA sequencing and RT‐PCR to select the targeted mice. To mitigate the impact of background genetics in experiments, KO mice were crossed with in‐house C57BL/6 mice to obtain *Ms4a4a* ^±^ mice. These mice were then intercrossed to generate WT and KO breeders. Besides, KO mice were crossed with *APP/PS1* mice to obtain *APP/PS1;Ms4a4a* ^±^ mice. These mice were then crossed with *Ms4a4a* ^+/−^ mice to generate WT, KO, *APP/PS1* and *APP/PS1;*KO breeders.

*P2ry12^CreERT2^* mice were generated by insertion of IRES‐iCreERT2 cassette before the 3′ UTR of *P2ry12* gene. The donor DNA plasmid was delivered together with Cas9 mRNA and sgRNA (5′‐CAATGTAGAACATTACCCAAGGG‐3′) into C57BL/6 mouse zygotes via microinjection. The resulting offsprings were screened by PCR genotyping, DNA sequencing to select the targeted mice. The mice were crossed to *Ai95* reporter mice to obtain *P2ry12^CreERT2^;Ai95* mice for calcium imaging.

*Ms4a4a^fl/fl^* mice were generated with targeting vector containing the loxP sites inserted to flank exon 2 of the *Ms4a4a* gene. The donor DNA plasmid was delivered together with Cas9 mRNA and single guide RNA (sgRNA) (5′ ‐sgRNA1: 5′ ‐GAGTTCGGAATACACAAGTGTTGG‐3′; 3′‐sgRNA2: 5′‐ GGGTGCATGCAAGTGGTTAAAGG‐3′) into C57BL/6 mouse zygotes via microinjection. The resulting offsprings were screened by PCR genotyping and DNA sequencing to select the targeted mice. *Ms4a4a^fl/fl^* mice were bred with *P2ry12^CreERT2^* mice to generate control (*Ms4a4a^fl/fl^*) and constitutive microglia‐specific *Ms4a4a* knockouts (*P2ry12^CreERT2^;Ms4a4a^fl/fl^*).

*Ms4a4a^HA/HA^* mice were generated by insertion of HA tag after the initiation codon of *Ms4a4a* gene. The donor DNA plasmid was delivered together with Cas9 mRNA and single guide RNA (sgRNA) (5′‐sgRNA: 5′‐ GCTGCCATGTTAGTCATACAAGG‐3′) into C57BL/6 mouse zygotes via microinjection. The resulting offspring were screened by PCR genotyping and DNA sequencing to select the targeted mice. The mice were bred in house to produce the littermates, which were randomly assigned to experimental groups.

### Acquisition of L929 Cell Supernatant

To obtain the supernatant of L929, cells should be passaged when they reach 80% confluence to avoid overgrowth. After expanding to the desired number of dishes, 5 mL of fresh complete culture medium was added to each 10 cm culture dish for further incubation. After 7 d, the supernatant was collected and filtered through a 0.2 µm filter. The filtrate was then aliquoted and stored in a −80 °C freezer.

### Culture of Bone Marrow‐Derived Macrophages

To cultivate BMDMs, mice were first euthanized by cervical dislocation, and their carcasses were thoroughly disinfected by immersion in 75% alcohol. The skin of the mouse's thigh was then cut open using surgical scissors, the muscle was torn apart, and one side of the femur and tibia were carefully removed. The muscles were stripped from the leg bones using tweezers, and the bones were washed multiple times in sterile PBS to ensure cleanliness. A diagonal cut was made at the top of the leg bone, and the bone marrow was flushed out multiple times with culture medium using a 1 mL syringe needle connected to a 5 mL syringe. The flushed contents were collected in complete culture medium containing 10% L929. The collected medium was then filtered through a 70 µm filter mesh to remove any debris. The cells obtained from this process were seeded at an appropriate density into 10 cm non‐adherent culture dishes and incubated at 37 °C with 5% CO₂. After seven days, the cells were scraped off with a cell scraper, counted, and reseeded at an appropriate density into well plates for further experimentation.

### Mouse Microglia and Astrocytes Isolation and Culture

Primary mouse microglia and astrocytes were obtained from P0‐P1 C57BL/6 WT mice. Pups were euthanized, soaked with 75% alcohol and were carefully decapitated. Brains were collected with clean sterile scissors and placed in a 10 cm dish containing 10 L iced dissociation medium DMEM (Cat. No. 10013136, Corning). All meninges were removed under a dissecting microscope. Brains were mechanically dissociated in dissociation medium. Dissociated cells were filtered through a 70 µm cell strainer and centrifuged at 500×*g* for 5 min at room temperature. Pellets were resuspended with culture medium (DMEM supplemented with 10% fetal bovine serum and 1% P/S) and plated at a density of three brains per T‐75 plastic culture flask (Thermo Fisher Scientific). The culture medium was changed into DMEM supplemented with 10% L929, 10% fetal bovine serum, and 1% P/S 72 h after isolation. One week later the flasks were shaken at 150 rpm using an orbital shaker for 1.5 h at 37 °C to collect microglia, for another 12 h at 37 °C with 5% CO_2_ and then 0.25% trypsin digestion was performed to obtain astrocytes. Microglia and astrocytes were collected in trizol for RT‐qPCR. If the microglia were used for culturing purposes, they were maintained at 37 °C in a humidified incubator with 5% CO_2_ for 12 h prior to use. In the wet experiments of this paper, the microglia mentioned are all cultured primary microglia, which did not involve sorting.

### Phagocytosis Assays

Stimuli, including fluorescent beads with diameters of 1 µm (Cat. No. L1030, Sigma), 2 µm (Cat. No. L4530, Sigma), and 6 µm (Zhichuan, Jiangsu), as well as GFP‐labeled *E. coli* (*E.coli* ^GFP^) and GFP‐labeled yeast (H99^GFP^), were added to cultured primary microglia at a quantity ratio of 10:1. After incubation for 2 h, the wells were rinsed five times with cold PBS to remove excess stimuli. The collected cells were then used for flow cytometry analysis. Cells that had phagocytosed the stimuli were detected based on their corresponding fluorescent signals, and the phagocytic effect was analyzed by assessing the intensity and proportion of these signals.

### Mitochondrial Respiration

OCR from cultured primary mouse microglia was measured using a Seahorse XFe‐96 Analyzer (Agilent). 100000 cells were seeded per well in 96‐well Seahorse plate. One hour before the Seahorse experiment, microglia were washed and incubated with the assay media (Agilent Seahorse XF Base Medium, 102353‐100, supplemented with 10 × 10^−3^ m glucose, 2 × 10^−3^ m l‐glutamine, 1 × 10^−3^ m sodium pyruvate, pH = 7.4) with or without adding 2 µm beads (Cat. No. L4530, Sigma) to cultured primary microglia in a non‐CO_2_ incubator. The beads were added at a quantity ratio of 10:1 (beads to microglia). Oligomycin (final concentration = 1.5 × 10^−6^ m, ab141829, Abcam), FCCP (final concentration = 1 × 10^−6^ m, C2920, Sigma) and a mixture of Rotenone (final concentration = 0.5 × 10^−6^ m, R8875, Sigma) and Antimycin A (final concentration = 0.5 × 10^−6^ m, ab141904, Abcam) were sequentially injected into the media. OCR was normalized to cell intensity.

### Ex Vivo Brain Slice Acquisition

Mice (8–10 weeks of age) were deeply anesthetized with avertin for perfusion by ice‐cold cutting buffer (120 × 10^−3^ m choline chloride, 2.6 × 10^−3^ m KCl, 1.25 × 10^−3^ m NaH_2_PO_4_, 26 × 10^−3^ m NaHCO_3_, 15 × 10^−3^ m glucose, 7 × 10^−3^ m MgCl_2_, and 0.5 × 10^−3^ m CaCl_2_, bubbled with 95% oxygen and 5% CO_2_ with an adjusted pH of 7.35–7.42 and an osmolarity of 280–300 mOsm kg^−1^). After decapitation, the head was immediately removed into ice‐cold cutting buffer. Coronal sections with a thickness of 300 µm were obtained using a vibratome (Leica VT1200S). Slices were rapidly transferred to a recovery chamber containing 34 °C aCSF (126 × 10^−3^ m NaCl, 3 × 10^−3^ m KCl, 1.2 × 10^−3^ NaH_2_PO_4_, 26 × 10^−3^ m NaHCO_3_, 10 × 10^−3^ m glucose, 1.3 × 10^−3^ m MgCl_2_, and 2.4 × 10^−3^ m CaCl_2_, bubbled with 95% oxygen and 5% CO_2_ with an adjusted pH of 7.35–7.42 and an osmolarity of 290–310 mOsm kg^−1^). Slices were incubated at 34 °C for 30 min then allowed to return to room temperature to use. The ex vivo brain slices used for supernatant collection were cultured in well plates. After adding 10 × 10^−3^ m PTZ for stimulation, the medium was changed after 30 s to avoid potential complications from prolonged PTZ exposure. The slices were then cultured for an additional 15 min before the supernatant was collected.

### Microglia Calcium Imaging

*Calcium Indicator Rhod‐2 for Calcium Imaging*: Cultured primary microglia were washed twice with Hanks’ balanced salt solution (HBSS) buffer (1×HBSS with 1.26 × 10^−3^ m Ca^2+^ and 10 × 10^−3^ m HEPES) before being loaded with 2 × 10^−6^ m calcium indicator Rhod‐2 acetoxymethyl ester (R1245MP, Thermo Fisher Scientific) for 30 min at 37 °C. Then, cells were washed twice and maintained in 200 µL HBSS buffer for live cell imaging. After basal Ca^2+^ concentration was monitored, the cells were added stimuli separately including HiLyteTM Fluor 488 Aβ^1‐42^ (Cat. No. AS‐65627, AnaSpec) and beads with diameters of 6 µm (Cat. No. 07312, Polysciences) during imaging. The stimuli were in excess. Rhod‐2 fluorescence was measured using PerkinElmer Opera phenix. The images and movies were generated using Nikon analysis software. For the kinetics data, the Rhod‐2 fluorescence intensity was normalized to the basal intensity.

*Cultured Primary Microglia* (*P2ry12^CreERT2^;Ai95*) *for Calcium Imaging*: Newborn pups (P0‐P1) positive for Ai95 were used for primary microglia culture. Three days after isolation, when the medium was changed, 1 × 10^−6^ m of 4‐hydroxytamoxifen was added to induce the expression of Ai95 in P2ry12 cells. After one week, the microglia culture was complete, and Ai95 signals in microglia could be detected by confocal microscopy to indicate changes in calcium flux. The beads used for stimulation had diameters of 6 µm (Cat. No. 07312, Polysciences).

*Ex Vivo Brain Slice (P2ry12^CreERT2^;Ai95) for Calcium Imaging: P2ry12^CreERT2^;Ai95* mice (8–10 weeks of age) were subjected to ex vivo brain slice culture one week after tamoxifen administration, using the method described above. After resting at room temperature for 30 min, the brain slices were ready for calcium imaging.

All calcium imaging was performed under a Nikon AXR NSPARC confocal microscope (Nikon, Japan).

*RT‐qPCR*: Total RNA for tissues or cultured cells was extracted by Trizol (AidLab) and reverse transcribed with PrimeScript RT reagent Kit with gDNA Eraser (Takara). Quantitative PCR (qPCR) was performed in StepOnePlus Real‐Time PCR system (ThermoFisher) using primers spanning introns.

### Cell Staining

The cultured primary microglia were first washed in cold PBS and then fixed in 1% PFA for 10 min at room temperature. After washing again in PBS, cells were permeabilized in PBST and blocked in 5% FBS in PBST at room temperature for 30 min. Cells were then stained by Flotillin‐1 (Cat. No. 18634, RRID:AB_2773040, Cell Signaling Technology) and MS4A4A (home‐made) with or without adding 2 µm beads (Cat. No. 19814, Polysciences) at a quantity ratio of 10:1 at 4 °C for overnight. Cells were washed three times in PBST and were then incubated with fluorescent secondary antibodies at room temperature for 1 h. Phalloidine (CA1620, Solarbio) was added to the cells and incubated at 4 °C for 1 h.

### Tissue Staining

The freshly excised human brain tissues were fixed with 1% PFA at 4 °C overnight, dehydrated in 30% sucrose and then embedded in OCT/30% sucrose media. The tissues were then processed for 14–16 µm cryosections. The sections were immunolabeled with indicated primary antibodies for Iba1 (Cat. No. ab178846, RRID:AB_2636859, Abcam) and NeuN (Cat. No. MAB377, RRID:AB_2298772, Millipore) at 4 °C for overnight and corresponding Alexa dye‐conjugated secondary antibodies at room temperature for 2 h. Slides were imaged under Nikon AXR NSPARC confocal microscope (Nikon, Japan).

### NIS Elements Rendering

Three‐dimensional renderings of DAPI/NeuN/Iba1 interactions were performed by imaging a representative z stack with a 100× objective (oil, NA:1.4) and a 0.33 mm step size on a Nikon AXR NSPARC confocal microscope. Rendering was performed in NIS elements. Iba1 microglia and NeuN neurons were manually surface rendered.

### Thio‐S Staining

The slides were dried at 50 °C for 10 min, washed with PBS for 5 min, dehydrated with 20% ethanol for 2 min, dehydrated with 40% ethanol for 2 min, dehydrated with 50% ethanol for 2 min, stained with 0.5% Thio‐s (in 50% ethanol) in the dark for 10 min, washed with 50% ethanol three times for 5 min each, rehydrated with 20% ethanol for 2 min, washed with PBS for 10 min.

### Western Blotting

Cell lysates were prepared on wet ice (4 °C) by homogenizing the sample in a cocktail of 1% LMNG buffer and protease and phosphatase inhibitors (Thermo‐Scientific, USA). Each sample was resolved on 10%–12% SDS‐PAGE. Following protein transfer to a PVDF membrane, then blocked for 1 h at room temperature with 5% skim milk. The incubation of the membrane with primary antibodies (diluted 1:1000 in 5% skim milk) was carried out for 12–16 h at 4 °C in rolling or shaking conditions. After several washes by PBST (0.1% Tween in PBS), the secondary antibody (diluted 1:10000 in 5% skim milk) was applied for 1 h. The membranes were incubated with chemiluminescence reagents (ThermoFisher) according to the manufacturer's instructions and ultrasensitive chemiluminescence imager (GE Amersham Imager600) was used to view the binding of antibodies.

### HA‐Immunoprecipitation and Mass Spectrometry Detection

To perform HA immunoprecipitation and mass spectrometry detection, an adequate amount of BMDMs was first cultured using the method described above. The BMDMs were then gently scraped off the culture dish using a cell scraper and collected into a 1.5 mL centrifuge tube, followed by centrifugation at 500*g* for 5 min. After discarding the supernatant, the pellet was resuspended in 1 mL of PBS buffer containing 1% LMNG (lauryl maltoside nonyl phenyl glycol ether) and 100× protease inhibitors. The sample was incubated in a rotating culture device at 4 °C for 2 h, then centrifuged at 14000*g* for 30 min. Anti‐HA magnetic beads were added to the supernatant and mixed in a rotating culture device at 4 °C for 2 h. The beads were washed four times with PBS buffer containing 0.002% LMNG. During the last wash, the beads were resuspended in an equal volume of 8 m urea and boiled at 37 °C for 10 min. The sample was then centrifuged at 14000*g* for 5 min, and the supernatant was carefully collected. The supernatant was split into two parts: half for WB to evaluate the eluted samples, and the other for SDS‐PAGE separation, followed by Coomassie Brilliant Blue staining and destaining. The lanes were excised and placed in deionized water before being sent to the Protein Platform at Tsinghua University for subsequent mass spectrometry identification. Finally, the samples underwent trypsin digestion followed by liquid chromatography‐mass spectrometry (LC‐MS) analysis.

### Co‐immunoprecipitation (CO‐IP)

The expression plasmids were constructed using a one‐step cloning kit (Vazyme Biotech). The full‐length mouse MS4A4A (Uniprot: A0A087WRT7, 1–236aa) was codon‐optimized and cloned into the pCAG vector with a recombinant 12aa flexible linker and FLAG tag at the C‐terminus. The full‐length mouse Flotillin‐1 (Uniprot: O08917, 1–428aa) was codon‐optimized and cloned into the pCAG vector with a recombinant 7aa flexible linker and Strep‐II tag at the C‐terminus. The truncations, including FLAG‐MS4A4AΔN (39‐236aa), MS4A4AΔC (1‐187aa)‐FLAG, and Flotillin‐1‐NTD (1‐163aa)‐Strep, were introduced using a standard two‐step polymerase chain reaction.

HEK293F cells (Invitrogen) were cultured in SMM 293T‐II medium (Sino Biological Inc.) at 37 °C under 5% CO_2_ in a ZCZY‐CS8 shaker (Zhichu, 120 rpm). When cell density reached 2.0 × 10^6^ cells mL^−1^, the cells were transiently transfected with the plasmids and polyethylenimine (PEI) (Polysciences, MW = 25000). For MS4A4A and Flotillin‐1 co‐expression, FLAG‐tagged MS4A4A full‐length or truncations (empty vector as negative control) plasmid and Strep‐tagged Flotillin‐1 full‐length or NTD plasmid, 20 µg of each, were mixed with 80 µg PEI (1:1:4 quality ratio) in 2 mL fresh medium for 20 min before adding into 20 mL cell culture for transfection. The transfected cells were cultured for 60 h before harvesting.

The transfected HEK293F cells were harvested at 3000*g*, and resuspended to 2 mL in HEPES buffer (20 × 10^−3^ m HEPES, pH 7.4 and 150 × 10^−3^ m NaCl) with 1% final concentration LMNG (Anatrace) and protease inhibitor added. After incubation at 4 °C for 2 h, the sample was centrifuged at 20000*g* for 30 min. Anti‐FLAG G1 affinity resin (Genscript, 1:50, pre‐balanced in HEPES buffer) was added into the supernatant and mixed at 4 °C for 1 h. The beads were washed with 50 times volume of HEPES buffer with 0.002% LMNG for five times, and were eluted by the wash buffer containing 1 mg mL^−1^ FLAG peptide. The elution samples were assessed by western blot analysis.

The protein samples were electrophoresed on 4%–12% or 4%–20% Bis‐Tris SDS‐PAGE (Genscript) with MES running buffer. Proteins were transferred to PVDF membranes (Millipore) using the eBlot L1 protein transfer system (Genscript) with the standard program. The blotted PVDF membranes were blocked with 5% skimmed milk dissolved in PBST buffer (PBS buffer with 0.05% Tween‐20) for 1 h at room temperature, and then incubated with the primary antibodies (diluted in PBST buffer with 5% skimmed milk) at 4 °C overnight with gentle shaking. After washing in PBST buffer five times, 5 min each at room temperature, the membranes were incubated with the secondary antibodies (diluted in PBST buffer with 5% skimmed milk) for 1 h at room temperature with gentle shaking. The secondary antibodies were then removed, and the membranes were further washes with PBST buffer five times, 5 min each at room temperature. The membranes were treated with Clarity Western ECL Substrate (BioRad) and imaged using the Amersham Imager 600 imaging system (GE Healthcare).

### Polyclonal Antibody Production for Mouse MS4A4A

The fusion proteins of the N‐terminal and C‐terminal fragments of mouse MS4A4A were produced by *E. coli*, as antigens for immunization of six mice. This procedure was conducted in accordance with the protocols established by the Cold Spring Harbor Laboratory. One week after the third immunization, blood was taken via retro‐orbital collection. Polyclonal antibodies of mouse MS4A4A were detected and verified in the serum of two mice by western blot and immunohistochemical staining. Their serum will be used as anti‐mouse MS4A4A antibodies for subsequent experiments.

### Survival Curve and Seizure Video Monitor

Long‐term continuous survival curve records of AD mice (P0–P60) housed in their home cages with clear cage lids (two mice per cage) were collected. Cages were checked every 24 h to ensure proper air conduction, ample and accessible food and water, and to collect any mouse found dead. Video monitoring of sporadic deaths and spontaneous convulsive seizures were performed by digital video camera (Xiaomi, Yuntai version).

### Drug‐Induced Seizures and Behavioral Scoring

Method 1: GABA receptor antagonist pentylenetetrazol (PTZ) was prepared in sterile saline (0.9%) at a concentration of 4 mg mL^−1^ and injected intraperitoneally at a dose of 40 mg kg^−1^ to induce seizures. After PTZ injection, all mice were placed in a transparent cage for 20 min for behavioral seizure scoring.^[^ ^89^ ^]^ Method 2: PTZ was prepared in sterile saline (0.9%) at a concentration of 1 mg mL^−1^ and injected intraperitoneally at a dose of 10 mg kg^−1^ to induce seizures every 10 min until death. After PTZ injection, all mice were placed in a transparent cage for behavioral seizure scoring until death. The experimental design is illustrated in Figure 1I.^[^ ^90^ ^]^ Method 3: Kainic acid was prepared in sterile saline (0.9%) at a concentration of 2 mg mL^−1^ and injected intraperitoneally at a dose of 17.5 mg kg^−1^ to induce seizures. After KA injection, all mice were placed in a transparent cage for 150 min for behavioral seizure scoring. The modified version of the Racine Scale (RS) was used as follows: 0, normal activity; 1, rigid posture or immobility; 2, stiffened or tail extension; 3, rearing with facial and manual automatism a well as partial body clonus, including forelimb clonus; 4, rearing and falling; 5, tonic‐clonic seizures with loss of posture or bouncing; 6, death. Stage 1 and 2 were classified as non‐convulsive seizures, whereas stages 3–6 were convulsive seizures. The experimenters were blinded to the genotypes of the mice.^[^ ^91^ ^]^

### Electroencephalographic (EEG) Recordings

Mice were deeply anesthetized with isoflurane, and 2 × 2 microelectrodes (conductor, 0.15 mm diameter) were implanted over the whole cortices. Mice were allowed to recover for one week, and EEG activity and image recording were recorded daily for up to two weeks.

### Parenchymal Stereotaxic Injection

To perform parenchymal stereotaxic injection, mice were first deeply anesthetized and eye ointment was applied. The fur on the top of the head was shaved, and the area was disinfected with povidone‐iodine and 75% ethanol. The skin at the top of the head was then cut open with scissors to fully expose the skull. AAV9‐hSyn‐EGFP‐2A‐Cre‐WPRE (1 × 10^13^ vg mL^−1^, 0.3 µL) or AAV9‐hSyn‐EGFP‐3XFLAG‐WPRE (1 × 10^13^ vg mL^−1^, 0.3 µL) was injected into the brain parenchyma (from bregma: 2.0 mm posterior, 1.65 mm left, 1.5 mm ventral) at a rate of 0.06 µL min^−1^. After the injection, the needle was left in place for 5 min before being slowly rotated out. The skin that had been cut open was carefully sutured with surgical thread. The mice were then returned to the animal facility to recover for at least two weeks. The tissue at the injection site was collected for mRNA detection.

### Intracranial Injection and LNPs Delivery

The LNPs were provided by Rhegen Bio and delivered into each mouse via intracranial injection at a dose of 0.5 mg mL^−1^ mRNA, with a volume of 2.5 µL per mouse. To perform intracranial injection and deliver LNPs, mice were first deeply anesthetized and eye ointment was applied. The fur on the back of the neck was shaved, and the area was disinfected with povidone‐iodine and 75% ethanol. The skin and muscles at the back of the neck were then cut open with scissors to fully expose the cranial foramen. A 2.5 µL microinjection needle was held and inserted into the cranial foramen to a depth of one‐fifth of its length, and the drug was injected at a rate of 0.5 µL min^−1^. After the injection, the needle was left in place for 5 min before being slowly rotated out. The skin that had been cut open was carefully sutured with surgical thread. The mice were then returned to the animal facility to recover. At 48 h post‐injection, a drug‐induced seizure assay was performed.

### Human Brain Tissues

The human brain tissues were collected at Peking University First Hospital. The study was approved by the Medical Ethics Committee of the First Hospital of Peking University, Ethical Review Approval No. 2021 Scientific Research 075 and was supported by the National Natural Science Foundation of China (82071263). Informed consent was obtained from the children and their parents, and the guardians signed an informed consent form for the use of the children's surgical samples for the study. The protocol was approved by the Institutional Ethics Committee of Peking University First Hospital. The freshly excised human brain tissues were subsequently used for bulk RNA‐seq, RT‐qPCR, and single‐cell RNA‐seq.

### Bulk RNA‐seq

For FCD_2b patient samples, total RNA was extracted and libraries were prepared using kits from BGI genomics according to manufacturer instructions. Bulk RNA‐seq was performed by BGISEQ (BGI genomics). For TSC patient samples, RNA purification, reverse transcription, library construction, and sequencing were performed at Shanghai Majorbio Bio‐pharm Biotechnology Co., Ltd. (Shanghai, China) according to the manufacturer's instructions (Illumina, San Diego, CA). Differential expression analysis was performed using the DESeq2 or DEGseq. *p*‐values were calculated by two‐tailed, paired Student's *t*‐test, ns *p* > 0.05, *p ≤ 0.05, **p ≤ 0.01, ***p ≤ 0.001, ****p ≤ 0.0001.

### Single‐Cell Preparation

For patient samples, scRNA‐seq was performed by SeekGene. After harvested, tissues were washed in ice‐cold PBS and dissociated using SeekMate Tissue Dissociation Reagent Kit A Pro (SeekGene, K01801301) from SeekGene as instructions. DNase I (Sigma, 9003‐98‐9) treatment was optional according to the viscosity of the homogenate. Cell count and viability was estimated using Fluorescence Cell Analyzer (Countstar Rigel, S2) with AO/PI reagent after removal erythrocytes (Solarbio, R1010) and then debris and dead cells removal was decided to be performed or not (Miltenyi, 130‐109‐398/130‐090‐101). Finally fresh cells were washed twice in the RPMI1640 (Gibco, 11875119) and then resuspended at 1 × 10^6^ cells mL^−1^ in RPMI 1640 and 2% FBS (Gibco, 10100147C).

For mouse samples, scRNA‐seq was performed by Singleron. The fresh cerebral cortex tissues were stored in the sCelLiveTM Tissue Preservation Solution (Singleron) on ice after the surgery within 30 min. The specimens were washed with HBSS for three times, minced into small pieces, and then digested with 3 mL sCelLive Tissue Dissociation Solution (Singleron) by Singleron PythoN Tissue Dissociation System at 37 °C for 15 min. The cell suspension was collected and filtered through a 40‐µm sterile strainer. Afterward, the GEXSCOPE red blood cell lysis buffer (RCLB, Singleron) was added, and the mixture (Cell: RCLB = 1:2) was incubated at room temperature for 5–8 min to remove red blood cells. The mixture was then centrifuged at 300×*g* 4 °C for 5 min to remove supernatant and suspended softly with PBS. Finally, the samples were stained with Trypan Blue and the cell viability was evaluated microscopically.

### Single‐Cell RNA‐seq Library Construction and Sequencing

For SeekGene, single‐cell RNA‐Seq libraries were prepared using SeekOne Digital Droplet Single Cell 3′ library preparation kit (SeekGene, Catalog No. K00202). Briefly, appropriate number of cells were mixed with reverse transcription reagent and then added to the sample well in SeekOne chip S3. Subsequently, Barcoded Hydrogel Beads (BHBs) and partitioning oil were dispensed into corresponding wells separately in chip S3. After emulsion droplet generation reverse transcription were performed at 42 °C for 90 minutes and inactivated at 85 °C for 5 min. Next, cDNA was purified from broken droplet and amplified in PCR reaction. The amplified cDNA product was then cleaned, fragmented, end repaired, A‐tailed and ligated to sequencing adaptor. Finally, the indexed PCR were performed to amplified the DNA representing 3′ polyA part of expressing genes which also contained Cell Barcode and Unique Molecular Index. The indexed sequencing libraries were cleaned up with VAHTS DNA Clean Beads (Vazyme, N411‐01), analyzed by Qubit (Thermo Fisher Scientific, Q33226) and Bio‐Fragment Analyzer (Bioptic, Qsep400). The libraries were then sequenced on illumina NovaSeq 6000.

For Singleron, single‐cell suspensions (2 × 10^5^ cells mL^−1^) with PBS (HyClone) were loaded onto microwell chip using the Singleron Matrix Single Cell Processing System. Barcoding Beads were subsequently collected from the microwell chip, followed by reverse transcription of the mRNA captured by the Barcoding Beads and to obtain cDNA, and PCR amplification. The amplified cDNA was then fragmented and ligated with sequencing adapters. The scRNA‐seq libraries were constructed according to the protocol of the GEXSCOPE Single Cell RNA Library Kits (Singleron). Individual libraries were diluted to 4 × 10^−9^ m, pooled, and sequenced on Illumina novaseq 6000 with 150 bp paired end reads.

### Analysis of Single‐Cell Transcriptomic Data

The raw sequencing data was initially processed using Fastp to trim primer sequences and remove low‐quality bases.^[^ ^92^ ^]^ SeekOne Tools were utilized for sequence processing and alignment to the mouse GRCm38 reference genome, resulting in the generation of gene expression matrices. Next, Seurat (v4.3.0) in R (v4.0.4) was applied to filter out low‐quality cells.^[^ ^93^ ^]^ For human single‐cell analysis, cells with fewer than 500 or more than 7000 detected genes, fewer than 1000 counts, or a mitochondrial gene percentage greater than 5% were excluded. For mouse single‐cell analysis, cells with fewer than 350 or more than 5000 detected genes, fewer than 500 counts, or a mitochondrial gene percentage exceeding 15% were excluded. Doublets were identified and removed using DoubletFinder (DoubletRate = CellNumber × 8 × 10^−6^), and the remaining cells were retained for further analysis.^[^ ^94^ ^]^

After quality control and filtering, library size normalization was applied to each cell using the NormalizeData function. The top 2000 variable genes were identified with FindVariableFeatures. Subsequently, all libraries were integrated using FindIntegrationAnchors and IntegrateData with default CCA parameters, followed by regression with ScaleData.^[^ ^95^ ^]^ Dimensionality reduction was performed using principal component analysis (PCA), t‐distributed stochastic neighbor embedding (t‐SNE), and uniform manifold approximation and projection (UMAP). Cell clustering was conducted with FindClusters, using 20 dimensions at a resolution of 0.8 for human data and 50 dimensions at a resolution of 1 for mouse data. Cluster annotation was performed manually based on previously reported marker genes.

Differential expression analysis of clustered genes was performed using FindMarkers, with significance defined by an adjusted *p*‐value of less than 0.05. GO analysis was conducted using clusterProfiler (v3.18.1) in R.^[^ ^96^ ^]^ Visualization of the results was carried out using DimPlot, DotPlot, DoHeatmap, and GraphPad PRISM 9. Cell–cell communication was analyzed with CellChat (v1.6.1), incorporating corrections for protein–protein interaction (PPI) networks in R.^[^ ^97^ ^]^

### Statistical Analysis

The data were analyzed with GraphPad Prism. For the comparisons of two groups, *p*‐values were calculated by unpaired or paired two‐tailed Student's *t*‐test. Data with multiple variables were calculated by one‐way ANOVA. For the comparisons of four groups, two‐way ANOVA (genotype and treatment) and multiple comparisons test were applied on samples.

## Conflict of Interest

The authors declare no conflict of interest.

## Author Contributions

M.J., Q.L., J.C., and R.L. contributed equally to this work. M.J., J.C., R.L., and H.Z. performed and analyzed experiments. Q.L. carried out the analysis on human single‐cell seq data. M.J. carried out the analysis on human bulk RNA seq data. Y.S., M.L., and L.C. provided the human brain samples. J.Y. and Y.H. provided the LNP‐*Il4*. W.Z. directed the project, conceived the experiments, and provided supervision. The manuscript was written by W.Z. with assistance from M.J.

## Supporting information



Supporting Information

Supplemental Video 1

Supplemental Video 2

Supplemental Video 3

Supplemental Video 4

Supplemental Video 5

Supplemental Video 6

## Data Availability

All data supporting the findings of the present study are available within the paper or from the corresponding author upon request.

## References

[1] P. Scheltens , B. De Strooper , M. Kivipelto , H. Holstege , G. Chételat , C. E. Teunissen , J. Cummings , W. M. van der Flier , Lancet 2021, 397, 1577. 33667416 10.1016/S0140-6736(20)32205-4 PMC8354300

[2] M. A. G. Gilbert , N. Fatima , J. Jenkins , Nature 2024, 631, 913. 38987603 10.1038/s41586-024-07680-x PMC11269202

[3] J. J. Palop , L. Mucke , Nat. Rev. Neurosci. 2016, 17, 777. 27829687 10.1038/nrn.2016.141 PMC8162106

[4] T. D. Anastacio , N. Matosin , L. Ooi , Transl. Psychiatry 2022, 12, 257. 35732622 10.1038/s41398-022-02024-7 PMC9217953

[5] M. Pease , K. Gupta , S. L. Moshé , D. J. Correa , A. S. Galanopoulou , D. O. Okonkwo , J. Gonzalez‐Martinez , L. Shutter , R. Diaz‐Arrastia , J. F. Castellano , Nat. Rev. Neurol. 2024, 20, 298. 38570704 10.1038/s41582-024-00954-y PMC12257072

[6] W. A. Hauser , M. L. Morris , L. L. Heston , V. E. Anderson , Neurology 1986, 36, 1226. 3092131 10.1212/wnl.36.9.1226

[7] S. Ziyatdinova , K. Gurevicius , N. Kutchiashvili , Epilepsy Res. 2011, 94, 75. 21300523 10.1016/j.eplepsyres.2011.01.003

[8] A. J. Barbour , S. Gourmaud , E. Lancaster , X. Li , D. A. Stewart , K. F. Hoag , D. J. Irwin , D. M. Talos , F. E. Jensen , Brain 2024, 147, 2169. 38662500 10.1093/brain/awae126 PMC11146435

[9] J. J. Palop , J. Chin , E. D. Roberson , Neuron 2007, 55, 697. 17785178 10.1016/j.neuron.2007.07.025 PMC8055171

[10] R. Minkeviciene , S. Rheims , M. B. Dobszay , M. Zilberter , J. Hartikainen , L. Fülöp , B. Penke , Y. Zilberter , T. Harkany , A. Pitkänen , H. Tanila , J. Neurosci. 2009, 29, 3453. 19295151 10.1523/JNEUROSCI.5215-08.2009 PMC6665248

[11] E. Beghi , A. Carpio , L. Forsgren , D. C. Hesdorffer , K. Malmgren , J. W. Sander , T. Tomson , W. A. Hauser , Epilepsia 2010, 51, 671. 19732133 10.1111/j.1528-1167.2009.02285.x

[12] J. Baker , T. Libretto , W. Henley , A. Zeman , Front. Neurol. 2019, 10, 1266. 31866927 10.3389/fneur.2019.01266 PMC6904279

[13] J. Vöglein , I. Ricard , S. Noachtar , W. A. Kukull , M. Dieterich , J. Levin , A. Danek , J. Neurol. 2020, 267, 2941. 32488295 10.1007/s00415-020-09937-7 PMC7501095

[14] A. Goate , M.‐C. Chartier‐Harlin , M. Mullan , Nature 1991, 349, 704. 1671712 10.1038/349704a0

[15] R. Sherrington , E. I. Rogaev , Y. Liang , Nature 1995, 375, 754. 7596406 10.1038/375754a0

[16] E. Levy‐Lahad , W. Wasco , P. Poorkaj , D. M. Romano , J. Oshima , W. H. Pettingell , C.‐E. Yu , P. D. Jondro , S. D. Schmidt , K. Wang , A. C. Crowley , Y.‐H. Fu , S. Y. Guenette , D. Galas , E. Nemens , E. M. Wijsman , T. D. Bird , G. D. Schellenberg , R. E. Tanzi , Science 1995, 269, 973. 7638622 10.1126/science.7638622

[17] J. C. Lambert , A. Ramirez , B. Grenier‐Boley , C. Bellenguez , Mol. Psychiatry 2023, 28, 2716. 37131074 10.1038/s41380-023-02076-1 PMC10615767

[18] C. Bellenguez , F. Küçükali , I. E. Jansen , L. Kleineidam , S. Moreno‐Grau , N. Amin , A. C. Naj , R. Campos‐Martin , B. Grenier‐Boley , V. Andrade , P. A. Holmans , A. Boland , V. Damotte , S. J. van der Lee , M. R. Costa , T. Kuulasmaa , Q. Yang , I. de Rojas , J. C. Bis , A. Yaqub , I. Prokic , J. Chapuis , S. Ahmad , V. Giedraitis , D. Aarsland , P. Garcia‐Gonzalez , C. Abdelnour , E. Alarcón‐Martín , D. Alcolea , M. Alegret , et al., Nat. Genet. 2022, 54, 412. 35379992 10.1038/s41588-022-01024-z PMC9005347

[19] A. C. Naj , G. Jun , G. W. Beecham , L.‐S. Wang , B. N. Vardarajan , J. Buros , P. J. Gallins , J. D. Buxbaum , G. P. Jarvik , P. K. Crane , E. B. Larson , T. D. Bird , B. F. Boeve , N. R. Graff‐Radford , P. L. De Jager , D. Evans , J. A. Schneider , M. M. Carrasquillo , N. Ertekin‐Taner , S. G. Younkin , C. Cruchaga , J. S. K. Kauwe , P. Nowotny , P. Kramer , J. Hardy , M. J. Huentelman , A. J. Myers , M. M. Barmada , F. Y. Demirci , C. T. Baldwin , et al., Nat. Genet. 2011, 43, 436. 21460841 10.1038/ng.801 PMC3090745

[20] P. Hollingworth , D. Harold , R. Sims , A. Gerrish , J.‐C. Lambert , M. M. Carrasquillo , R. Abraham , M. L. Hamshere , J. S. Pahwa , V. Moskvina , K. Dowzell , N. Jones , A. Stretton , C. Thomas , A. Richards , D. Ivanov , C. Widdowson , J. Chapman , S. Lovestone , J. Powell , P. Proitsi , M. K. Lupton , C. Brayne , D. C. Rubinsztein , M. Gill , B. Lawlor , A. Lynch , K. S. Brown , P. A. Passmore , D. Craig , et al., Nat. Genet. 2011, 43, 429. 21460840 10.1038/ng.803 PMC3084173

[21] R. Sims , S. J. van der Lee , A. C. Naj , Nat. Genet. 2017, 49, 1373. 28714976 10.1038/ng.3916 PMC5669039

[22] R. Guerreiro , A. Wojtas , J. Bras , M. Carrasquillo , E. Rogaeva , E. Majounie , C. Cruchaga , C. Sassi , J. S. K. Kauwe , S. Younkin , L. Hazrati , J. Collinge , J. Pocock , T. Lashley , J. Williams , J.‐C. Lambert , P. Amouyel , A. Goate , R. Rademakers , K. Morgan , J. Powell , P. St George‐Hyslop , A. Singleton , J. Hardy , N. Engl. J. Med. 2013, 368, 117.23150934

[23] C. Bellenguez , C. Charbonnier , B. Grenier‐Boley , Neurobiol. Aging 2017, 59, 220. 10.1016/j.neurobiolaging.2017.07.001 28789839

[24] H. Neumann , M. J. Daly , N. Engl. J. Med. 2013, 368, 182. 23151315 10.1056/NEJMe1213157

[25] T. Jonsson , H. Stefansson , S. Steinberg , I. Jonsdottir , P. V. Jonsson , J. Snaedal , S. Bjornsson , J. Huttenlocher , A. I. Levey , J. J. Lah , D. Rujescu , H. Hampel , I. Giegling , O. A. Andreassen , K. Engedal , I. Ulstein , S. Djurovic , C. Ibrahim‐Verbaas , A. Hofman , M. A. Ikram , C. M. van Duijn , U. Thorsteinsdottir , A. Kong , K. Stefansson , N. Engl. J. Med. 2013, 368, 107. 23150908 10.1056/NEJMoa1211103 PMC3677583

[26] S. Hong , L. Dissing‐Olesen , B. Stevens , Curr. Opin. Neurobiol. 2016, 36, 128. 26745839 10.1016/j.conb.2015.12.004 PMC5479435

[27] S. Wang , R. Sudan , V. Peng , Y. Zhou , S. Du , C. M. Yuede , T. Lei , J. Hou , Z. Cai , M. Cella , K. Nguyen , P. L. Poliani , W. L. Beatty , Y. Chen , S. Cao , K. Lin , C. Rodrigues , A. H. Ellebedy , S. Gilfillan , G. D. Brown , D. M. Holtzman , S. Brioschi , M. Colonna , Cell 2022, 185, 4153. 36306735 10.1016/j.cell.2022.09.033 PMC9625082

[28] C. Y. D. Lee , A. Daggett , X. Gu , Neuron 2018, 97, 1032. 29518357 10.1016/j.neuron.2018.02.002 PMC5927822

[29] T. K. Ulland , W. M. Song , S. C.‐C. Huang , J. D. Ulrich , A. Sergushichev , W. L. Beatty , A. A. Loboda , Y. Zhou , N. J. Cairns , A. Kambal , E. Loginicheva , S. Gilfillan , M. Cella , H. W. Virgin , E. R. Unanue , Y. Wang , M. N. Artyomov , D. M. Holtzman , M. Colonna , Cell 2017, 170, 649. 28802038 10.1016/j.cell.2017.07.023 PMC5573224

[30] Y. Liang , T. R. Buckley , L. Tu , S. D. Langdon , T. F. Tedder , Immunogenetics 2001, 53, 357. 11486273 10.1007/s002510100339

[31] Y. Liang , T. F. Tedder , Genomics 2001, 72, 119. 11401424 10.1006/geno.2000.6472

[32] I. Mattiola , A. Mantovani , M. Locati , Trends Immunol. 2021, 42, 764. 34384709 10.1016/j.it.2021.07.002

[33] G. K. Arthur , L. C. Ehrhardt‐Humbert , D. B. Snider , Cell. Signalling 2020, 71, 109617. 32240745 10.1016/j.cellsig.2020.109617 PMC7293826

[34] I. Mattiola , F. Tomay , M. De Pizzol , R. Silva‐Gomes , B. Savino , T. Gulic , A. Doni , S. Lonardi , M. Astrid Boutet , A. Nerviani , R. Carriero , M. Molgora , M. Stravalaci , D. Morone , I. N. Shalova , Y. Lee , S. K. Biswas , G. Mantovani , M. Sironi , C. Pitzalis , W. Vermi , B. Bottazzi , A. Mantovani , M. Locati , Nat Immunol. 2019, 20, 1012. 31263276 10.1038/s41590-019-0417-y PMC7176488

[35] Y. Li , Z. Shen , Z. Chai , Y. Zhan , Y. Zhang , Z. Liu , Y. Liu , Z. Li , M. Lin , Z. Zhang , W. Liu , S. Guan , J. Zhang , J. Qian , Y. Ding , G. Li , Y. Fang , H. Deng , Gut 2023, 72, 2307. 37507218 10.1136/gutjnl-2022-329147 PMC10715532

[36] S. F. You , L. Brase , F. Filipello , A. K. Iyer , J. Del‐Aguila , J. He , R. D’Oliveira Albanus , J. Budde , J. Norton , J. Gentsch , N. M. Dräger , S. M. Sattler , M. Kampmann , L. Piccio , J. C. Morris , R. J. Perrin , E. McDade , S. M. Paul , A. G. Cashikar , B. A. Benitez , O. Harari , C. M. Karch , Dominantly Inherited Alzheimer Network , medRxiv. 2023, 10.1101/2023.02.06.23285545.

[37] B. De Strooper , Cell 2016, 164, 603. 26871627 10.1016/j.cell.2015.12.056

[38] O. Lazarov , J. Robinson , Y.‐P. Tang , I. S. Hairston , Z. Korade‐Mirnics , V. M.‐Y. Lee , L. B. Hersh , R. M. Sapolsky , K. Mirnics , S. S. Sisodia , Cell 2005, 120, 701. 15766532 10.1016/j.cell.2005.01.015

[39] G. S. Green , M. Fujita , H.‐S. Yang , M. Taga , A. Cain , C. McCabe , N. Comandante‐Lou , C. C. White , A. K. Schmidtner , L. Zeng , A. Sigalov , Y. Wang , A. Regev , H.‐U. Klein , V. Menon , D. A. Bennett , N. Habib , P. L. De Jager , Nature 2024, 633, 634. 39198642 10.1038/s41586-024-07871-6 PMC11877878

[40] M. Xu , D. F. Zhang , R. Luo , Alzheimer's Dementia 2018, 14, 215. 10.1016/j.jalz.2017.08.012 28923553

[41] K. E. Reyes‐Marin , A. Nunez , Brain Res. 2017, 1677, 93. 28963050 10.1016/j.brainres.2017.09.026

[42] Y. Huang , Cell 2012, 148, 1204. 22424230 10.1016/j.cell.2012.02.040 PMC3319071

[43] S. S. Harris , F. Wolf , B. De Strooper , M. A. Busche , Neuron 2020, 107, 417. 32579881 10.1016/j.neuron.2020.06.005

[44] L. François , A. Romagnolo , M. J. Luinenburg , J. J. Anink , P. Godard , M. Rajman , J. van Eyll , A. Mühlebner , A. Skelton , J. D. Mills , S. Dedeurwaerdere , E. Aronica , Nat. Commun. 2024, 15, 2180. 38467626 10.1038/s41467-024-46592-2 PMC10928184

[45] S. Wullschleger , R. Loewith , M. N. Hall , Cell 2006, 124, 471. 16469695 10.1016/j.cell.2006.01.016

[46] R. A. Saxton , D. M. Sabatini , Cell 2017, 168, 960. 28283069 10.1016/j.cell.2017.02.004 PMC5394987

[47] O. Devinsky , A. Vezzani , T. J. O'Brien , N. Jette , I. E. Scheffer , M. de Curtis , P. Perucca , Nat. Rev. Dis. Primers 2018, 4, 18024. 29722352 10.1038/nrdp.2018.24

[48] A. Deczkowska , H. Keren‐Shaul , A. Weiner , M. Colonna , M. Schwartz , I. Amit , Cell 2018, 173, 1073. 29775591 10.1016/j.cell.2018.05.003

[49] R. Sanyal , M. J. Polyak , J. Zuccolo , M. Puri , L. Deng , L. Roberts , A. Zuba , J. Storek , J. M. Luider , E. M. Sundberg , A. Mansoor , E. Baigorri , M. P. Chu , A. R. Belch , L. M. Pilarski , J. P. Deans , Immunol. Cell Biol. 2017, 95, 611. 28303902 10.1038/icb.2017.18

[50] M. Prinz , S. Jung , Cell 2019, 179, 292. 31585077 10.1016/j.cell.2019.08.053

[51] M. W. Salter , B. Stevens , Nat. Med. 2017, 23, 1018. 28886007 10.1038/nm.4397

[52] M. Colonna , O. Butovsky , Annu. Rev. Immunol. 2017, 35, 441. 28226226 10.1146/annurev-immunol-051116-052358 PMC8167938

[53] D. He , H. Xu , H. Zhang , Immunity 2022, 55, 159. 34982959 10.1016/j.immuni.2021.12.001 PMC9074730

[54] X. Zhao , Y. Liao , S. Morgan , Cell Rep. 2018, 22, 2080. 29466735 10.1016/j.celrep.2018.02.004 PMC5880308

[55] J. Park , Y. Choi , E. Jung , S. H. Lee , J. W. Sohn , W. S. Chung , EMBO J. 2021, 40, 107121. 10.15252/embj.2020107121 PMC8327958 34013588

[56] A. D. Umpierre , B. Li , K. Ayasoufi , W. L. Simon , S. Zhao , M. Xie , G. Thyen , B. Hur , J. Zheng , Y. Liang , D. B. Bosco , M. A. Maynes , Z. Wu , X. Yu , J. Sung , A. J. Johnson , Y. Li , L.‐J. Wu , Neuron 2024, 112, 1959. 38614103 10.1016/j.neuron.2024.03.017 PMC11189754

[57] D. C. Patel , B. P. Tewari , L. Chaunsali , H. Sontheimer , Nat. Rev. Neurosci. 2019, 20, 282. 30792501 10.1038/s41583-019-0126-4 PMC8558781

[58] A. Badimon , H. J. Strasburger , P. Ayata , Nature 2020, 586, 417. 32999463 10.1038/s41586-020-2777-8 PMC7577179

[59] M. D. Bootman , K. Rietdorf , H. Hardy , Y. Dautova , E. Corps , C. Pierro , E. Stapleton , E. Kang , D. Proudfoot , Encycl. Life Sci. 2012, 10.1002/9780470015902.a0001265.pub3.

[60] M. J. Berridge , M. D. Bootman , H. L. Roderick , Nat. Rev. Mol. Cell Biol. 2003, 4, 517. 12838335 10.1038/nrm1155

[61] Z. Fu , R. MacKinnon , Proc. Natl. Acad. Sci. USA 2024, 121, 2409334121. 10.1073/pnas.2409334121 PMC11260169 38985763

[62] Y. Sui , W. Zeng , Eur. J. Immunol. 2020, 50, 1602. 32589266 10.1002/eji.202048585

[63] Z. Czimmerer , T. Varga , S. Poliska , I. Nemet , A. Szanto , L. Nagy , Immunobiology 2012, 217, 1301. 22954708 10.1016/j.imbio.2012.08.270

[64] C. R. Jack , D. A. Bennett , K. Blennow , Alzheimer's Dementia 2018, 14, 535. 10.1016/j.jalz.2018.02.018 PMC5958625 29653606

[65] S. Gourmaud , D. A. Stewart , D. J. Irwin , Brain 2022, 145, 324. 34264340 10.1093/brain/awab268 PMC9126019

[66] A. A. Horvath , A. Papp , J. Zsuffa , A. Szucs , J. Luckl , F. Radai , F. Nagy , Z. Hidasi , G. Csukly , G. Barcs , A. Kamondi , Clin. Neurophysiol. 2021, 132, 1982. 34034963 10.1016/j.clinph.2021.03.050

[67] K. A. Vossel , M. C. Tartaglia , H. B. Nygaard , A. Z. Zeman , B. L. Miller , Lancet Neurol. 2017, 16, 311. 28327340 10.1016/S1474-4422(17)30044-3 PMC5973551

[68] A. Caccamo , A. Magri , D. X. Medina , E. V. Wisely , M. F. López‐Aranda , A. J. Silva , S. Oddo , Aging Cell 2013, 12, 370. 23425014 10.1111/acel.12057 PMC3655115

[69] S. F. Kazim , S. C. Chuang , W. Zhao , R. K. Wong , R. Bianchi , K. Iqbal , Front. Aging Neurosci. 2017, 9, 71. 28392767 10.3389/fnagi.2017.00071 PMC5364175

[70] A.‐L. Lin , J. B. Jahrling , W. Zhang , N. DeRosa , V. Bakshi , P. Romero , V. Galvan , A. Richardson , J. Cereb. Blood Flow Metab. 2017, 37, 217. 26721390 10.1177/0271678X15621575 PMC5167110

[71] A. L. Lin , W. Zheng , J. J. Halloran , J. Cereb. Blood Flow Metab. 2013, 33, 1412. 23801246 10.1038/jcbfm.2013.82 PMC3764385

[72] K. Princen , T. Van Dooren , M. van Gorsel , N. Louros , X. Yang , M. Dumbacher , I. Bastiaens , K. Coupet , S. Dupont , E. Cuveliers , A. Lauwers , M. Laghmouchi , T. Vanwelden , S. Carmans , N. Van Damme , H. Duhamel , S. Vansteenkiste , J. Prerad , K. Pipeleers , O. Rodiers , L. De Ridder , S. Claes , Y. Busschots , L. Pringels , V. Verhelst , E. Debroux , M. Brouwer , S. Lievens , J. Tavernier , M. Farinelli , et al., Science 2024, 384, add6260. 10.1126/science.add6260 PMC11827694 38815015

[73] Y. Fang , X. Si , J. Wang , Z. Wang , Y. Chen , Y. Liu , Y. Yan , J. Tian , B. Zhang , J. Pu , Neurology 2023, 101, 399. 10.1212/WNL.0000000000207423 PMC10435057 37225432

[74] D. B. Bosco , V. Kremen , K. Haruwaka , S. Zhao , L. Wang , B. A. Ebner , J. Zheng , M. Xie , A. Dheer , J. F. Perry , A. Barath , A. T. Nguyen , G. A. Worrell , L.‐J. Wu , Brain, Behav., Immun. 2025, 123, 540. 39353548 10.1016/j.bbi.2024.09.034 PMC11924143

[75] Y. F. F. Deming , F. Cignarella , C. Cantoni , S. Hsu , R. Mikesell , Z. Li , J. L. Del‐Aguila , U. Dube , F. G. Farias , J. Bradley , J. Budde , L. Ibanez , M. V. Fernandez , K. Blennow , H. Zetterberg , A. Heslegrave , P. M. Johansson , J. Svensson , B. Nellgård , A. Lleo , D. Alcolea , J. Clarimon , L. Rami , J. L. Molinuevo , M. Suárez‐Calvet , E. Morenas‐Rodríguez , G. Kleinberger , M. Ewers , O. Harari , C. Haass , et al., Sci. Transl. Med. 2019, 11, eaau2291. 31413141 10.1126/scitranslmed.aau2291 PMC6697053

[76] R. L. Winfree , E. Nolan , L. Dumitrescu , K. Blennow , H. Zetterberg , K. A. Gifford , K. R. Pechman , M. Seto , V. A. Petyuk , Y. Wang , J. Schneider , D. A. Bennett , A. L. Jefferson , T. J. Hohman , Mol. Neurodegener. 2024, 19, 41. 38760857 10.1186/s13024-024-00727-7 PMC11101336

[77] K. De Vlaminck , H. Van Hove , D. Kancheva , I. Scheyltjens , A. R. Pombo Antunes , J. Bastos , M. Vara‐Perez , L. Ali , M. Mampay , L. Deneyer , J. F. Miranda , R. Cai , L. Bouwens , D. De Bundel , G. Caljon , B. Stijlemans , A. Massie , J. A. Van Ginderachter , R. E. Vandenbroucke , K. Movahedi , Immunity 2022, 55, 2085. 36228615 10.1016/j.immuni.2022.09.005

[78] A. B. DePaula‐Silva , Viruses 2024, 16, 119. 38257819 10.3390/v16010119 PMC10819099

[79] A. Silvin , J. Qian , F. Ginhoux , Cell Mol. Immunol. 2023, 20, 1277. 37365324 10.1038/s41423-023-01053-6 PMC10616292

[80] A. Silvin , S. Uderhardt , C. Piot , S. Da Mesquita , K. Yang , L. Geirsdottir , K. Mulder , D. Eyal , Z. Liu , C. Bridlance , M. S. Thion , X. M. Zhang , W. T. Kong , M. Deloger , V. Fontes , A. Weiner , R. Ee , R. Dress , J. W. Hang , A. Balachander , S. Chakarov , B. Malleret , G. Dunsmore , O. Cexus , J. Chen , S. Garel , C. A. Dutertre , I. Amit , J. Kipnis , F. Ginhoux , Immunity 2022, 55, 1448. 35931085 10.1016/j.immuni.2022.07.004

[81] F. H. Brennan , Y. Li , C. Wang , A. Ma , Q. Guo , Y. Li , N. Pukos , W. A. Campbell , K. G. Witcher , Z. Guan , K. A. Kigerl , J. C. E. Hall , J. P. Godbout , A. J. Fischer , D. M. McTigue , Z. He , Q. Ma , P. G. Popovich , Nat. Commun. 2022, 13, 4096. 35835751 10.1038/s41467-022-31797-0 PMC9283484

[82] G. Cruse , M. A. Beaven , S. C. Music , P. Bradding , A. M. Gilfillan , D. D. Metcalfe , Mol. Biol. Cell 2015, 26, 1711. 25717186 10.1091/mbc.E14-07-1221 PMC4436782

[83] K. Farber , H. Kettenmann , Glia 2006, 54, 656. 17006894 10.1002/glia.20412

[84] T. Moller , Glia 2002, 40, 184. 12379906 10.1002/glia.10152

[85] B. Brawek , B. Schwendele , K. Riester , K. Riester , S. Kohsaka , C. Lerdkrai , Y. Liang , O. Garaschuk , Acta Neuropathol. 2014, 127, 495. 24407428 10.1007/s00401-013-1242-2

[86] A. D. Umpierre , L. L. Bystrom , Y. Ying , Y. U. Liu , G. Worrell , L. J. Wu , Elife 2020, 9, 56502. 10.7554/eLife.56502 PMC7402678 32716294

[87] K. Sharma , L. J. Wu , U. B. Eyo , Trends Neurosci. 2020, 43, 197. 32209451 10.1016/j.tins.2020.01.008

[88] J. A. French , M. Bebin , M. A. Dichter , Epilepsia Open 2021, 6, 483. 34270884 10.1002/epi4.12526 PMC8408600

[89] M. Putra , S. Puttachary , G. Liu , G. Lee , T. Thippeswamy , Front. Cell Neurosci. 2020, 14, 592374. 33363455 10.3389/fncel.2020.592374 PMC7752812

[90] M. T. Haile , S. Khoja , G. de Carvalho , R. F. Hunt , L. Y. Chen , Transl. Psychiatry 2023, 13, 97. 36941261 10.1038/s41398-023-02394-6 PMC10027846

[91] S. Jain , N. Nirwan , N. B. Agarwal , D. Vohora , Neuromethods 2021, 167, 121.

[92] S. Chen , Y. Zhou , Y. Chen , J. Gu , Bioinformatics 2018, 34, i884. 30423086 10.1093/bioinformatics/bty560 PMC6129281

[93] Y. Hao , S. Hao , E. Andersen‐Nissen , W. M. Mauck , S. Zheng , A. Butler , M. J. Lee , A. J. Wilk , C. Darby , M. Zager , P. Hoffman , M. Stoeckius , E. Papalexi , E. P. Mimitou , J. Jain , A. Srivastava , T. Stuart , L. M. Fleming , B. Yeung , A. J. Rogers , J. M. McElrath , C. A. Blish , R. Gottardo , P. Smibert , R. Satija , Cell 2021, 184, 3573. 34062119 10.1016/j.cell.2021.04.048 PMC8238499

[94] C. S. McGinnis , L. M. Murrow , Z. J. Gartner , Cell Syst. 2019, 8, 329. 30954475 10.1016/j.cels.2019.03.003 PMC6853612

[95] A. Butler , P. Hoffman , P. Smibert , E. Papalexi , R. Satija , Nat. Biotechnol. 2018, 36, 411. 29608179 10.1038/nbt.4096 PMC6700744

[96] G. Yu , L. G. Wang , Y. Han , Q. Y. He , Omics 2012, 16, 284. 22455463 10.1089/omi.2011.0118 PMC3339379

[97] S. Jin , C. F. Guerrero‐Juarez , L. Zhang , I. Chang , R. Ramos , C.‐H. Kuan , P. Myung , M. V. Plikus , Q. Nie , Nat. Commun. 2021, 12, 1088. 33597522 10.1038/s41467-021-21246-9 PMC7889871

