# Supplementary material for: Microglial MS4A4A Protects against Epileptic Seizures in Alzheimer's Disease
Source: Adv Sci (Weinh). 2025 May 11;12(22):2417733. doi: 10.1002/advs.202417733 (PMC12165070; doi:10.1002/advs.202417733)
Supplement: Supplementary file 1 — Supporting Information [file ADVS-12-2417733-s005.doc]

**Supporting Information**

Supporting Information is available from the Wiley Online Library or from the author.

**TABLE OF CONTENTS**

The study has unveiled significant new insights into the role of MS4A4A in Alzheimer's disease-related epilepsy, highlighting its impact on microglial phagocytosis, mitochondrial metabolism, and cytoskeleton, and demonstrating its therapeutic potential in epilepsy management.

*
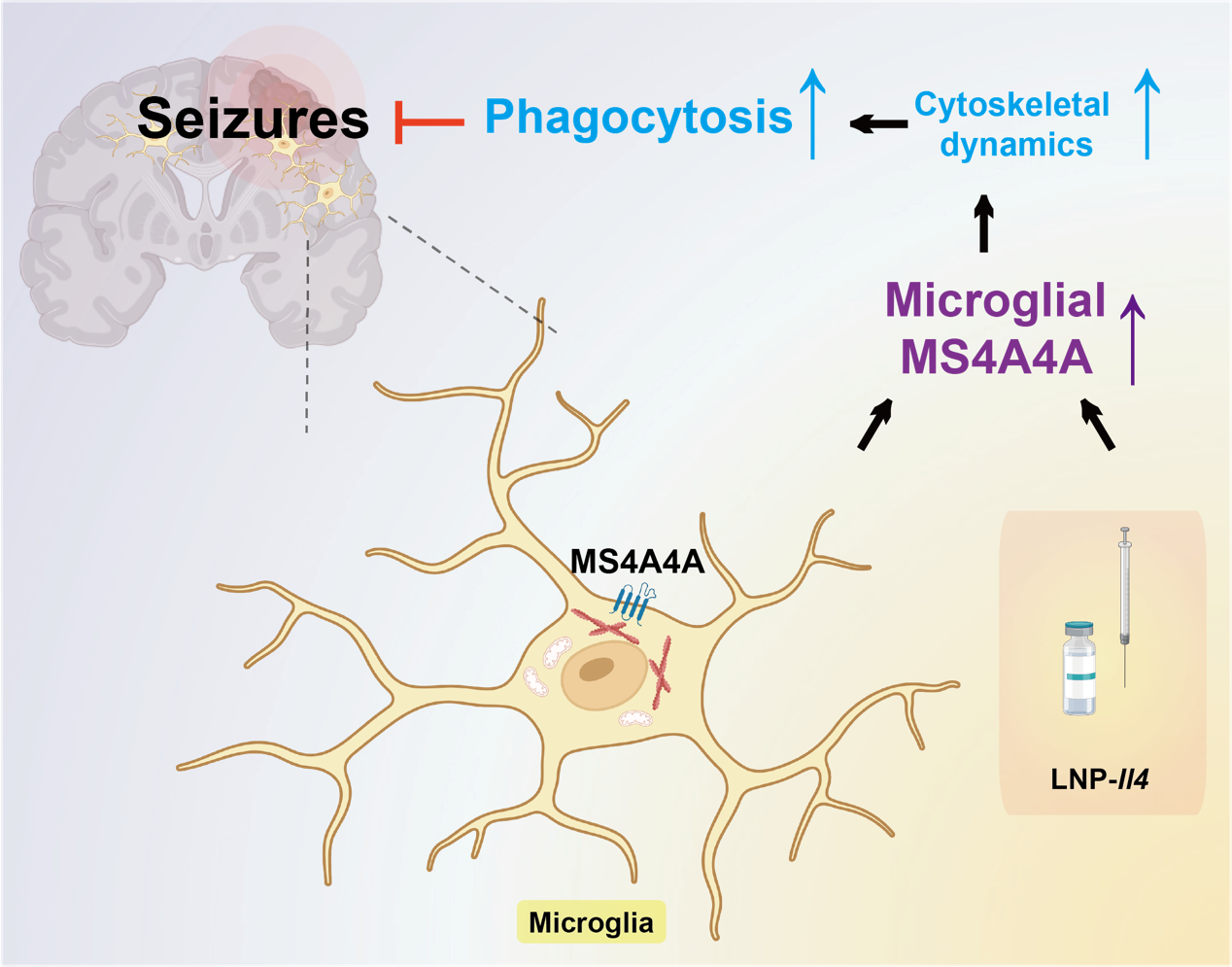
*

Supporting Information

**Microglial MS4A4A protects against epileptic seizures in Alzheimer's disease**

*Meng Jiang, Qingqing Li, Jianhui Chen, Ruochong Li, Jun Yao, Yong Hu, Haizheng Zhang, Lixin Cai^*^, Maoguo Luo^*^, Yu Sun^*^, Wenwen Zeng^*^*

**Figure S1. The validation of CRISPR/Cas9-edited mice and assessment of the epilepsy in global KO mice**


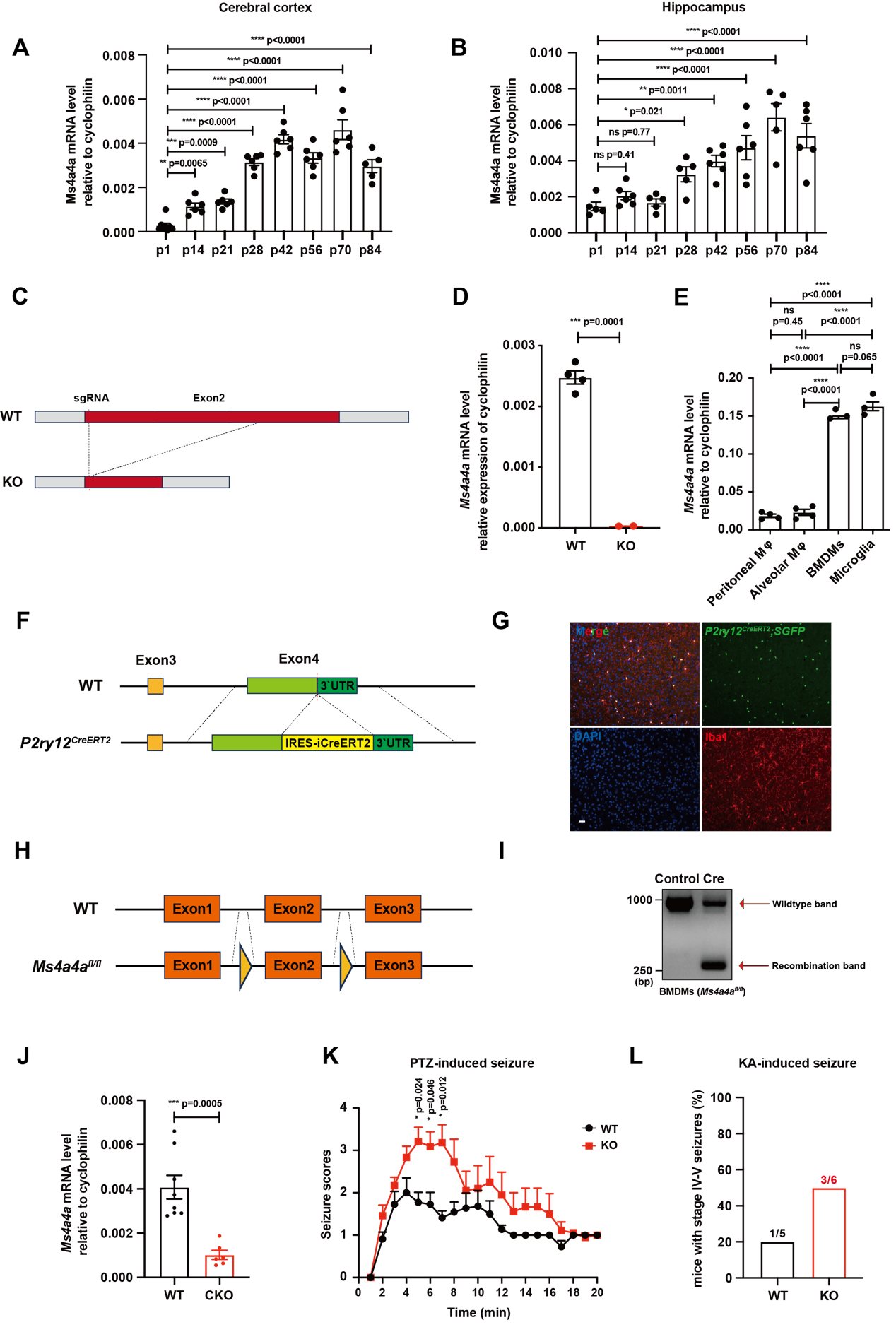
A, B) Relative transcript levels of *Ms4a4a* in cerebral cortex (A), hippocampus (B) of P1-P84 WT mice determined by RT-qPCR. n ≥ 5. C) Strategy of KO mouse. D) Relative transcript levels of *Ms4a4a* in KO mouse determined by RT-qPCR. n ≥ 2. E) Relative transcript levels of *Ms4a4a* in peritoneal macrophages, alveolar macrophages, BMDMs, and microglia determined by RT-qPCR. n ≥ 3. F, G) Strategy of *P2ry12^CreERT2^* mouse (F) and efficiency verification by immunohistochemical staining (G). Scale bar, 10 μm. H, I) Strategy of *Ms4a4a^fl/fl^* mouse (H) and efficiency verification by pMX-IRES-Cre infected BMDMs (I). J) Relative transcript levels of *Ms4a4a* in *P2ry12^CreERT2^;Ms4a4a^fl/fl^* mouse. n ≥ 6. K, L) Time course of behavioral status epilepticus during the initial 20 minutes following PTZ administration in WT and KO mice (K). The proportion of stage IV-V seizures of WT and KO mice injected by KA within 150 minutes (L). n ≥ 3. p values were calculated by one-way ANOVA (A, B and E) and two-tailed, unpaired Student’s *t*-test (D, J and K), ns p > 0.05, *p ≤ 0.05, **p ≤ 0.01, ***p ≤ 0.001, ****p ≤ 0.0001.

**Figure S2. Cellular landscape with *IL4* signaling in TSC**


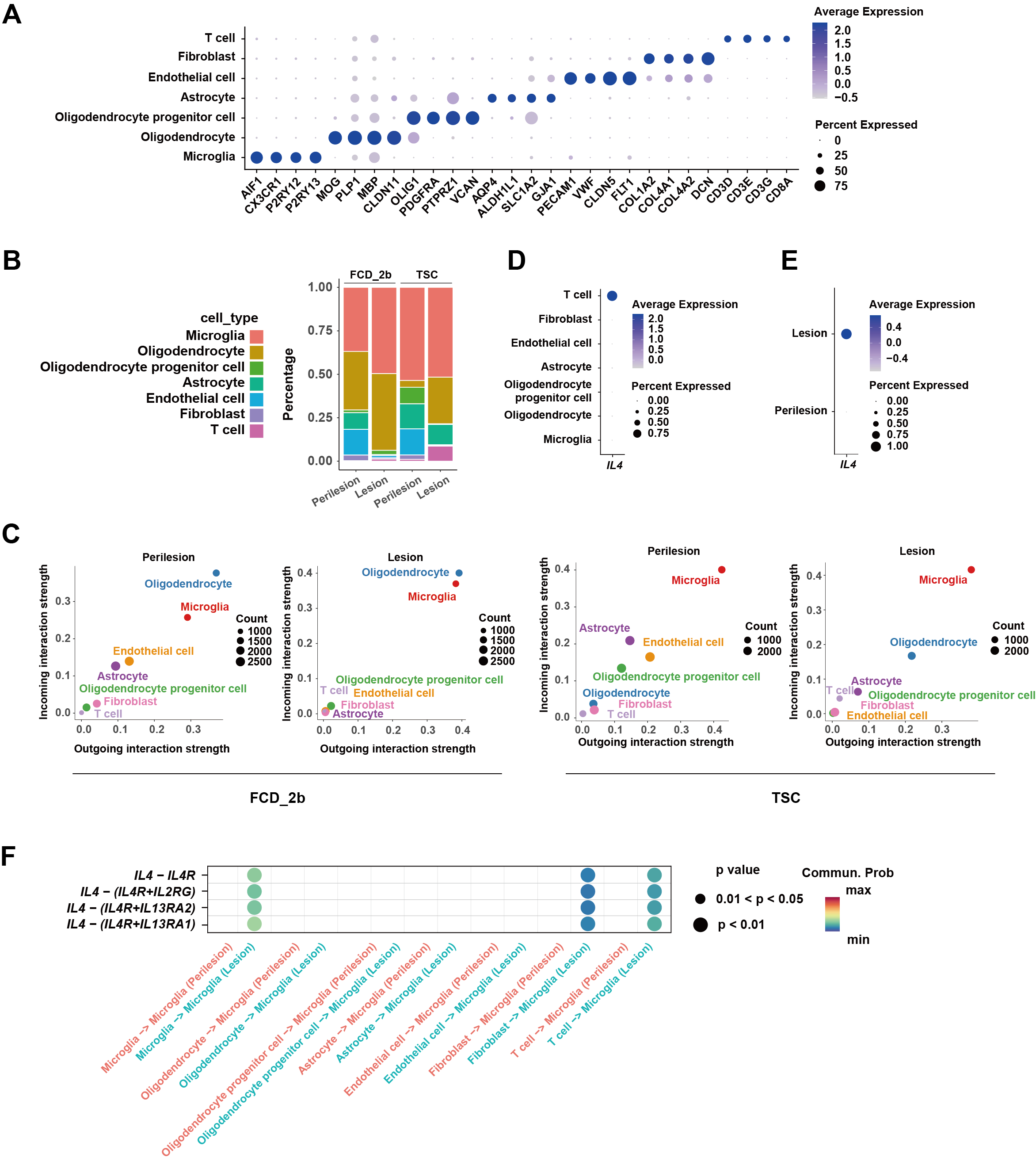
A) Dotplot showing marker genes of 7 clusters. The size of the dots represents the percentage of the expression in all cell types, and the color of the dots represents the degree of enrichment. B) The percentage of 7 cell types in lesional and perilesional tissues of FCD_2b and TSC are shown. C) Incoming and outgoing interaction strength in lesional and perilesional tissues of FCD_2b and TSC. D, E) Dotplot showing the expression of *IL4* across 7 clusters (D) and the expression profile of the *IL4* in T cells (E). F) The intercellular signaling between *IL4* and its receptors.

**Figure S3. Distinct microglial functions in AD mouse models upon *Ms4a4a* deletion: implications for phagocytosis and cytoskeletal dynamics**

**
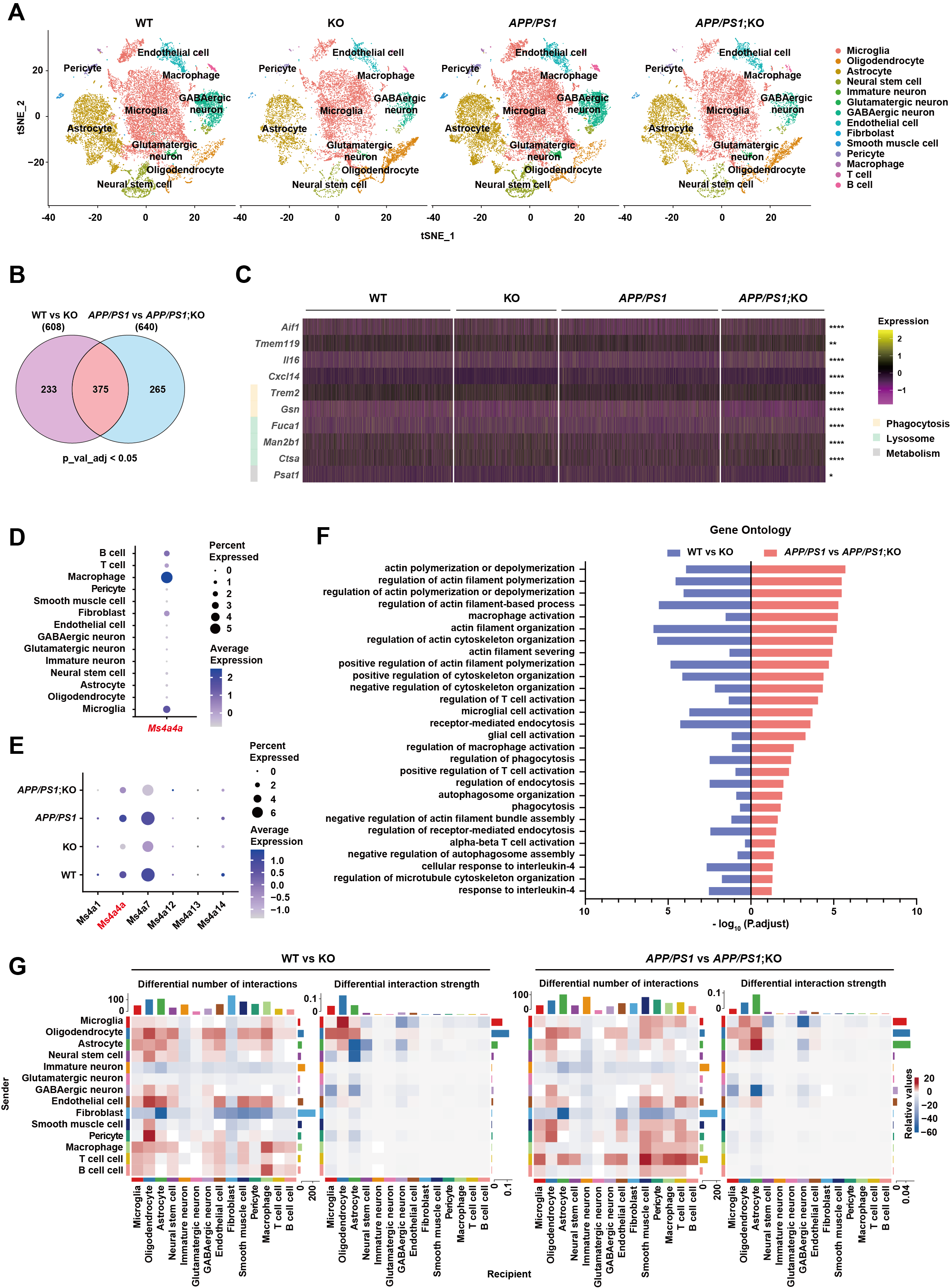
** A) Uniform Manifold Approximation and Projection (UMAP) plot of 14 clusters. B) Venn diagram showing the differential gene numbers of WT vs KO and *APP/PS1* vs *APP/PS1*;KO. p_val_adj < 0.05. C) Heatmap depicting the relative expression of inflammatory genes, phagocytosis genes, lysosome genes, and metabolic genes in microglia of WT, KO, *APP/PS1* and *APP/PS1;*KO. The star on the right indicates the p values between *APP/PS1* and *APP/PS1*;KO. D, E) Dotplot showing the expression of *Ms4a4a* across 14 clusters (D) and the expression profile of the *Ms4a* family in microglia (E). F) Bar chart showing selectively enriched GO terms of WT vs KO and *APP/PS1* vs *APP/PS1*;KO. G) Heatmap showing the total number of interactions and strength of interactions between cell types in WT vs KO and *APP/PS1* vs *APP/PS1*;KO groups. p values were calculated by Wilcoxon Rank Sum test (B, C), *p ≤ 0.05, **p ≤ 0.01, ****p ≤ 0.0001.

**Figure S4. Phagocytic defects caused by *Ms4a4a* knockout is not substrate-specific**

**
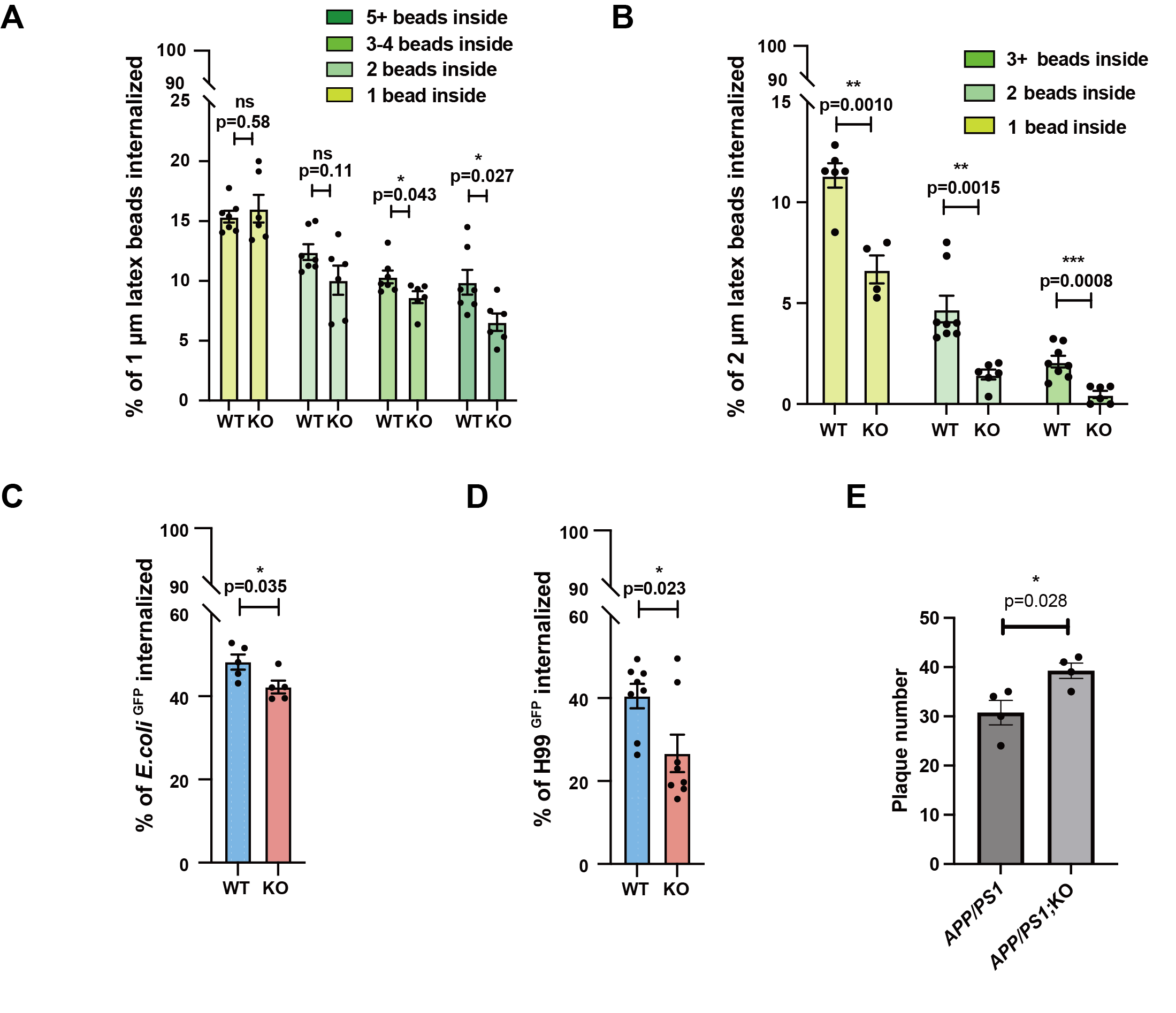
**

A-D) Upon engulfing fluorescently labeled phagocytic materials, the microglia became fluorescent and were detectable by flow cytometry instruments. The phagocytic ability of KO microglia, as indicated by the proportion of 1 µm beads inside, was found to decline significantly in the group where the number of beads per cell was greater than or equal to 3, compared to the WT microglia (A). The phagocytic ability of KO microglia, as indicated by the proportion of 2 µm beads (B), *E.coli*^GFP^ (C), and H99^GFP^ (D) inside, was found to decline significantly. E) The number of plaque stained by Thio-S in *APP/PS1* and *APP/PS1;*KO mice assessed in corresponding cerebral cortex sections. Survey of 4 slides of the same location from 4 mice per group. p values were calculated by two-tailed, unpaired Student’s t-test (A-E), ns p > 0.05, *p ≤ 0.05, **p ≤ 0.01, ***p ≤ 0.001.

**Figure S5. MS4A4A and Flotillin-1 are colocalized during bead phagocytosis**


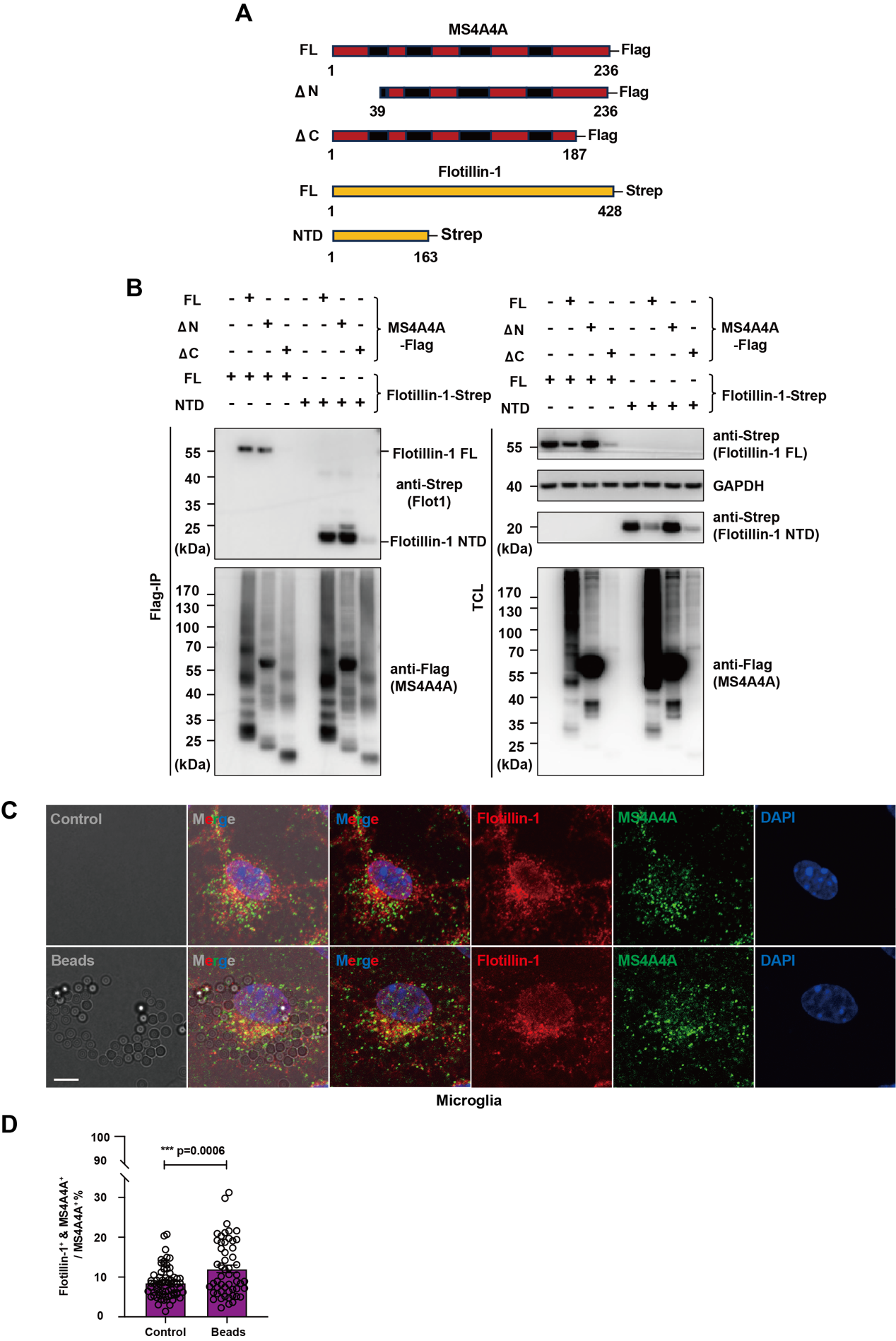
A, B) Schematic diagram of MS4A4A, Flotillin-1 and their mutants (A). Co-immunoprecipitation assays using overexpressed MS4A4A-Flag and Flotillin-1-Strep proteins, along with their truncated variants, expressed in 293F cells (B). C, D) Representative immunofluorescence images showing the spatial position of MS4A4A and Flotillin-1 under the two-hour stimuli with beads in cultured primary microglia (C). Flotillin-1^+^ & MS4A4A^+^ / MS4A4A^+^ ratio was analyzed (D). Scale bar, 5 μm. Survey of over 50 microglia from more than six subfields per group. Data are presented as mean ± SEM. p values were calculated by two-tailed, unpaired Student’s *t*-test (D), ***p ≤ 0.001.

**Figure S6. A proposed model for the suppressive role of microglial MS4A4A in seizures**


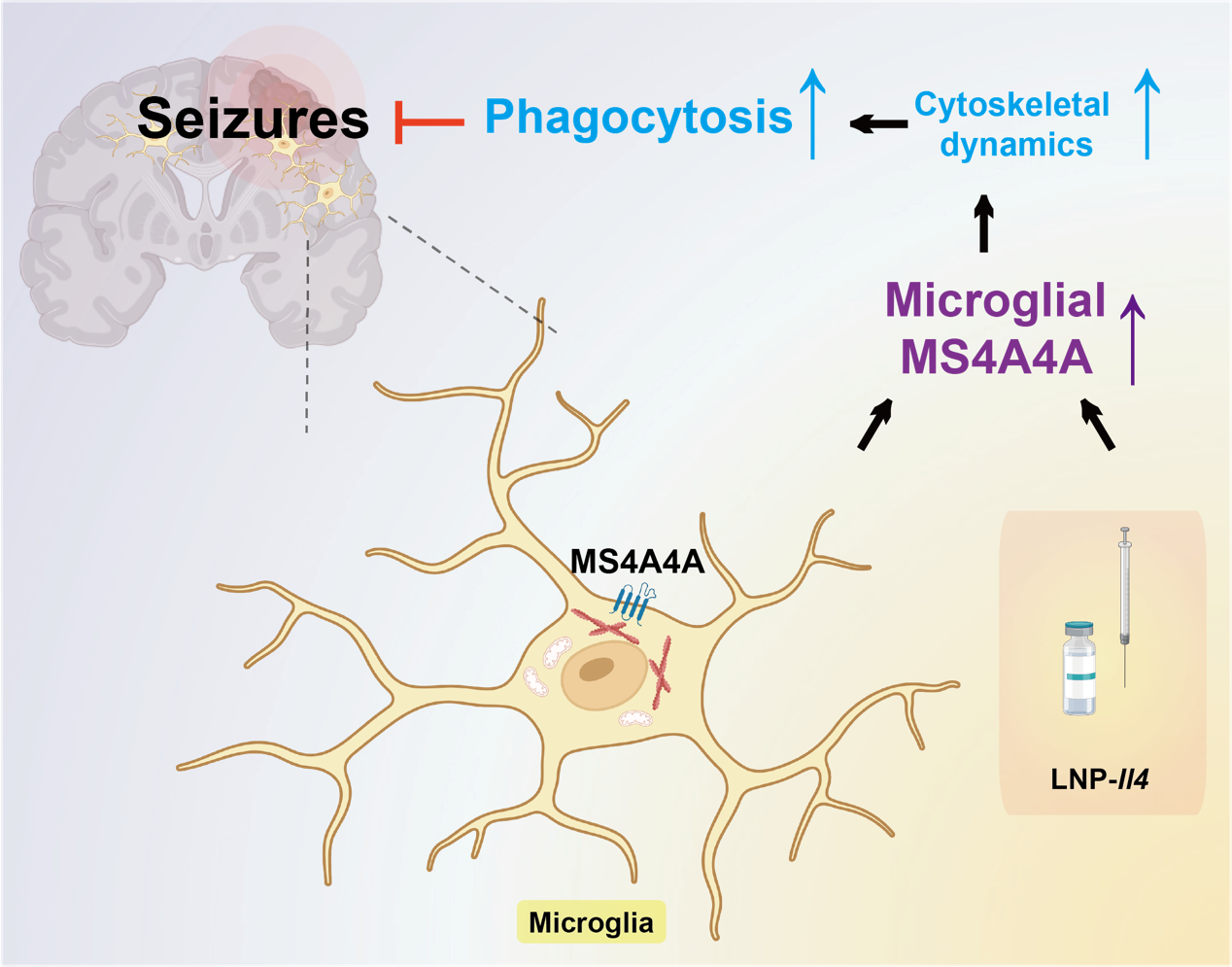
MS4A4A is significantly upregulated in microglia during seizures in both AD mice and epileptic patients. It enhances phagocytic activity and suppresses seizures. Inducing MS4A4A through central delivery of LNP-*Il4* alleviates seizure conditions.

**Video 1.** Fatal epileptic seizure.

**Video 2.** Calcium activity (ΔF/F) of cultured *P2ry12^CreERT2^;Ai95* microglia in the process of touching and engulfing 6 μm beads. Scale bar, 20 μm.

**Video 3.** Calcium activity (ΔF/F) of cultured WT and KO microglia indicated by calcium flux indicator Rhod2 after adding HiLyteTM Fluor 488 Aβ^1-42^. Scale bar, 20 μm.

**Video 4.** Calcium activity (ΔF/F) of cultured WT and KO microglia indicated by calcium flux indicator Rhod2 after adding 6 μm beads. Scale bar, 20 μm.

**Video 5.** Calcium activity (ΔF/F) of cultured WT and KO microglia indicated by calcium flux indicator Rhod2 after adding PBS. Scale bar, 20 μm.

**Video 6.** Calcium activity (ΔF/F) of live *P2ry12^CreERT2^;Ai95* brain slices after adding aCSF, 100 mM PTZ, and 100 mM KA separately. Scale bar, 5 μm.
